# Combined Theoretical and Experimental Investigations: Design, Synthesis, Characterization, and In Vitro Cytotoxic Activity Assessment of a Complex of a Novel Ureacellobiose Drug Carrier with the Anticancer Drug Carmustine

**DOI:** 10.3390/molecules29143359

**Published:** 2024-07-17

**Authors:** Marta Hoelm, Stanisław Porwański, Paweł Jóźwiak, Anna Krześlak

**Affiliations:** 1 Theoretical and Structural Group, Department of Physical Chemistry, Faculty of Chemistry, University of Lodz, Pomorska 163/165, 90-236 Lodz, Poland; 2 Department of Organic and Applied Chemistry, Faculty of Chemistry, University of Lodz, Tamka 12, 91-403 Lodz, Poland; stanislaw.porwanski@chemia.uni.lodz.pl; 3 Department of Cytobiochemistry, Faculty of Biology, University of Lodz, Pomorska 141/143, 90-236 Lodz, Poland; pawel.jozwiak@biol.uni.lodz.pl (P.J.); anna.krzeslak@biol.uni.lodz.pl (A.K.)

**Keywords:** carmustine, complexes with new drug carriers, DFT calculations, synthesis, cytotoxicity, drug delivery systems

## Abstract

Drug delivery systems (DDSs) are used to transport drugs which are characterized by some pharmaceutical problems to the specific target site, enhancing therapeutic efficacy and reducing off-target accumulation in the body. In this work, one of the recently synthesized molecules, 1,10-*N*,*N’*-bis-(β-ᴅ-ureidocellobiosyl)-4,7,13,16-tetraoxa-1,10-diazacyclooctadecane (TN), was tested as a potential drug carrier towards the anticancer drug carmustine. For this purpose, different techniques were used, from synthesis and calculations to cytotoxicity assessment. Our results showed that TN is characterized by a very compact geometry, which significantly impacts its complexation properties. Although it forms a very stable complex with carmustine, it adopts a non-inclusion geometry, as verified by both experimental and theoretical NMR analyses. The cytotoxicity study performed for all analyzed molecules (TN; carmustine; TN:carmustine complex) towards normal and cancer (breast and colon) cells revealed that TN is not toxic and that the formation of complexes with carmustine reduces the toxicity of carmustine to normal cells.

## 1. Introduction

Carmustine, also known as BCNU (1,3-bis(2-chloroethyl)-1-nitrosourea), is a chemotherapeutic agent belonging to the nitrosourea class of alkylating agents. It was synthesized in 1966 and approved a decade later by the U.S. Food and Drug Administration as an anticancer drug for the treatment of various malignancies, particularly brain tumors [1,2]. The mechanism of action of carmustine is based on its ability to form cross-links in DNA, subsequently interfering with the normal DNA replication process, preventing cancer cells from dividing and leading to their death [3]. The effective anticancer properties of BCNU primarily result from its lipophilic nature. According to Lipinski’s rule of five [4,5], lipophilicity is an important factor characterizing the physicochemical properties of a drug [6,7]. The logarithmic n-octanol-water partition coefficient (logP) is a descriptor of lipophilicity, and for carmustine, it is 1.53 [8,9,10]. Despite the fact that a drug should possess a logP value higher than 2 [11], BCNU crosses the blood–brain barrier [12,13]. This ability is undoubtedly also influenced by its low molecular weight [14,15] of approximately 214 Da [16]. A small molecular weight facilitates penetration not only into cancerous tissues but also into normal ones [17], which consequently results in side effects for patients. The most common adverse effects of carmustine include myelosuppression (decreased blood cell production) [18], nausea, vomiting, and an increased risk of infections. A long-term use or high doses of carmustine may also lead to postponed side effects, such as pulmonary fibrosis [18] or secondary malignancies. For patients, carmustine is most commonly administered intravenously, which is a fundamental method of drug delivery that bypasses absorption barriers and allows for the direct introduction of the drug into the circulation [19,20]. However, BCNU is poorly soluble in water [21], which adversely affects its bioavailability and necessitates proportionally higher dosages [14].

Drug delivery systems (DDSs) have been proposed as a remedy for the pharmaceutical challenges associated with carmustine. The primary goal of DDSs is to deliver carmustine to the target site and achieve the desired therapeutic effect. To date, various DDS formulations for BCNU have been developed. Notably, nanoformulations [13,22,23,24] such as carbon nano-onions [25], nanosheets [26,27], and nanomicelles [28] have been highlighted. Recent studies by Li et al. [28] demonstrated that the nanomicelle–BCNU formulation is more effective at killing glioma cancer cells than the traditional combination of BCNU and *O*^6^-BG (an AGT inhibitor). Other DDS compounds proposed for carmustine delivery include liposomes [18,29], hydrogels [30], and wafers [31,32]. Among these, the most notable is the wafer formulation, which has been approved by the FDA and is marketed under the trade name Gliadel [12,33]. However, it should be emphasized that the literature contains opposing opinions regarding the use of this preparation. For instance, the recent work of Roux et al. [34] indicated that BCNU wafer is safe and effective in treating cancer, provided that this formulation is applied after the surgical resection of supratentorial glioma. In turn, other studies [35,36,37,38] showed that the results regarding the survival rate after using the carmustine wafer are inconclusive and require further research.

**Figure 1:**
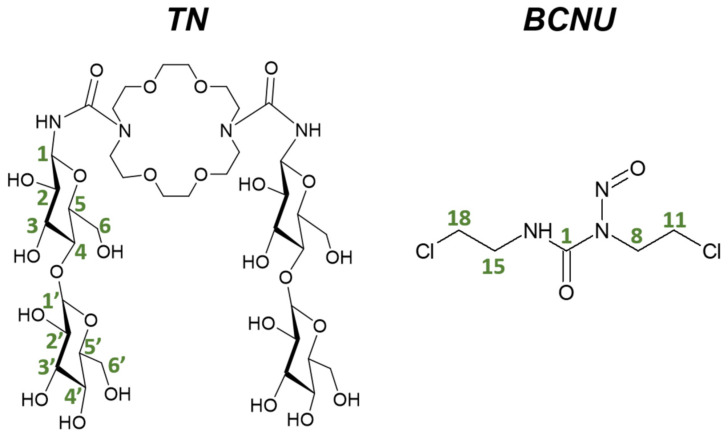
Structural formulas of 1,10-*N*,*N’*-bis-(β-ᴅ-ureidocellobiosyl)-4,7,13,16-tetraoxa-1,10-diazacyclooctadecane (TN) and carmustine (BCNU) molecules, along with the carbon atom numbering used in the NMR analysis.

The role of DDS may also be played by the cryptand named 1,10-*N*,*N’*-bis-(β-ᴅ-ureidocellobiosyl)-4,7,13,16-tetraoxa-1,10-diazacyclooctadecane (TN; Figure 1). It possesses a diazacrown ether and two cellobiose units. Crown ethers are known for their excellent complexation abilities towards various ions and neutral molecules [39,40], while sugars are highly selective transporters, often considered the most important fragments in drug delivery vehicles [41]. Although TN was synthetized some time ago [42], knowledge about it remains limited. Experimental studies show that it can form a stable complex with the anticancer drug busulfan in a 1:1 stoichiometry. Also, attempts were made to obtain a crystal structure, but so far, this has only been successful for its acetylated form [42].

In this paper, for the first time, a comprehensive experimental and theoretical characterization of the TN carrier and its complex with the anticancer drug carmustine is conducted. Synthesis and NMR analysis confirmed the successful formation of the carrier and its complex with BCNU. In addition, cytotoxicity studies were carried out to assess the effect of complex formation on carmustine cytotoxicity in non-tumoral and cancer cells. Theoretical studies provided insights into the stability of the complexes, the most dominant interactions, binding sites, and thermodynamic parameters. A theoretical NMR analysis was also performed and compared with the experimental outcomes. Together, these results contribute to a comprehensive understanding of the behavior of the drug carrier and its potential in carmustine delivery, offering valuable insights for further research in the field of drug delivery systems.

## 2. Results

### 2.1. Conformational Analysis of the Drug Carrier and Carmustine

**Figure 2:**
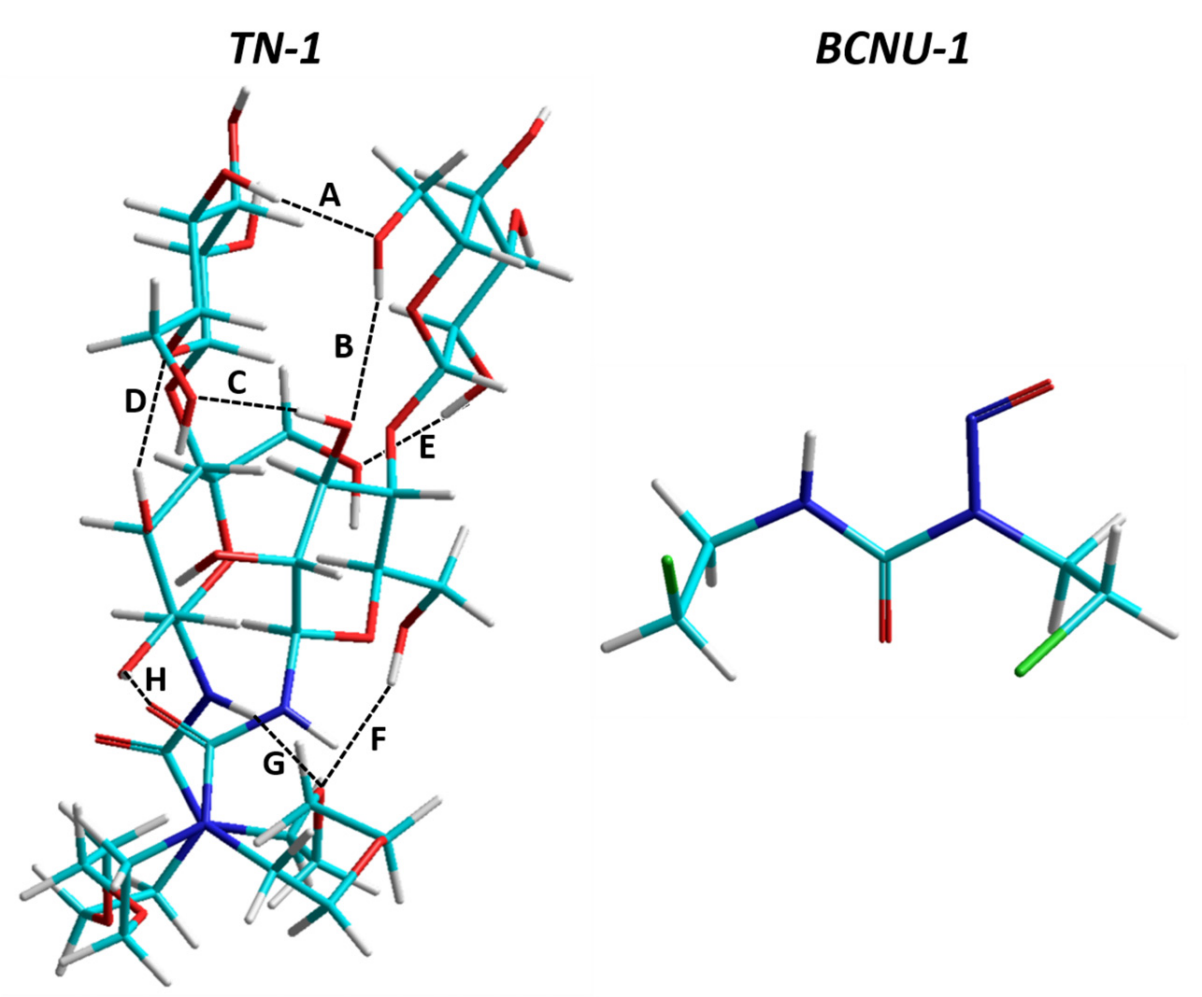
The most stable conformers of 1,10-*N*,*N’*-bis-(β-ᴅ-ureidocellobiosyl)-4,7,13,16-tetraoxa-1,10-diazacyclooctadecane (TN-1) and carmustine (BCNU-1) obtained from the M06-2X-GD3/6-31G(d,p) calculations in water (PCM). The dotted lines with letters indicate hydrogen bonds. Atom colors: carbon—cyan; oxygen—red; nitrogen—dark blue; hydrogen—gray; chlorine—green.

The conformational search performed for both isolated molecules, TN and BCNU, allows for obtaining the most energetically preferable structures, which are presented in Figure 2. Other, less stable configurations are shown in Figures S4 and S5 in the Electronic Supplementary Files (ESI). These configurations were selected from the final step of the conformational analysis performed at the M06-2X-GD3/6-31G(d,p) theory level in water (PCM). As can be seen in Figure 2, TN exhibits a very complex structure, with a small area between the ureidocellobiosyl units. In fact, this trend is observed in all conformers of TN. This is due to the formation of a large number of intramolecular hydrogen bonds (HBs), which, according to Jeffrey’s categorization [43], are moderate. They mainly form between the ureidocellobiose units; however, in the three most stable conformers (TN-1; TN-2; TN-3), the diazacrown ether is also involved in this interaction. TN-1 possesses eight HBs, which is the highest number, while other, less stable conformers have three to six HBs. Thus, it can be concluded that, to some extent, HBs play an important role in determining the geometry of TN. The parametrical geometries of the hydrogen bonds formed in TN are listed in Table S1.

In the case of carmustine, its geometry does not favor the formation of the intramolecular hydrogen bonds, at least not those of moderate strength. This does not imply that hydrogen bonds do not occur in the molecule; they may form, but considering the BCNU geometry, they are likely to be weak. Analyzing the crystal structure of BCNU [16], it can be seen that intramolecular hydrogen bonds are also not observed. However, BCNU forms intermolecular hydrogen bonds between neighboring molecules within unit cells. It is worth mentioning that the groups involved in these interactions are also involved in forming hydrogen bonds with TN in the most stable complexes (). A discussion of these interactions is provided below in the subsection *The configurational search, structural, and energetical parameters of the TN complexation process.*

As was mentioned above, the most stable conformers of both TN and BCNU are selected from the M06-2X-GD3/6-31G(d,p) calculations. However, the basis set used in the optimizations is rather small, and thus to verify the 6-31G(d,p) conformer ranking, single-point (SP) calculations were performed. SP calculations were conducted for the 200 lowest energetically conformers of TN using the same functional (M06-2X) but larger basis sets: 6-31++G(d,p) and 6-311++G(d,p). Since BCNU is much smaller than TN, re-optimizations were performed for all its 6-31G(d,p)-optimized structures using the same basis sets as for TN. The results of this analysis are shown for the 20 lowest energetically favorable structures in Figure S6. Generally, for both TN and BCNU, all methods indicate the same conformer as the most stable. The energy difference values (ΔE) calculated between the first and last conformer (regardless of the computational method) are significantly smaller in the case of carmustine, indicating that its potential energy surface is less corrugated.

**Figure 3:**
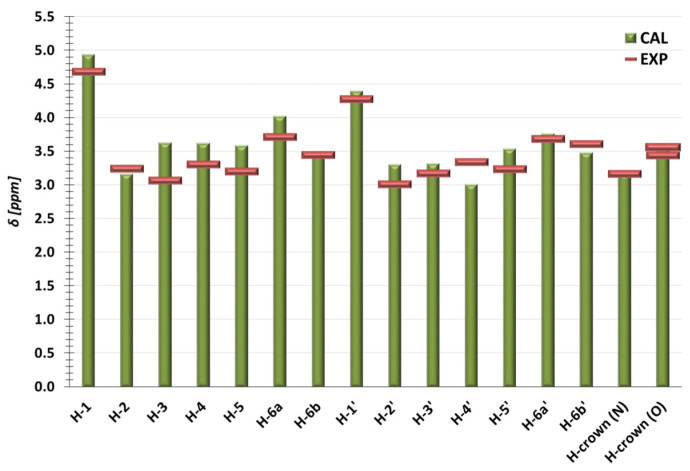
Comparison of the NMR chemical shifts δ (ppm) obtained for the drug carrier TN from the experimental measurements (EXP) performed in DMSO-d_6_ and from calculations (CAL) conducted in DMSO. H-6a and H-6b indicate the average of the higher and lower values of the H-6 chemical shifts, respectively. The same applies to H-6a’ and H-6b’.

Theoretical results aimed at finding the most stable conformer should be verified through comparison with experimental data. Attempts were made to obtain the crystal structure of TN; however, this was achieved only for its acetylated form [42]. Additionally, to the best of our knowledge, TN has not been theoretically studied, especially in terms of its structural parameters and geometry. Therefore, we made a comparison of the NMR chemical shifts obtained from the theoretical analysis and experimental measurements. The latter were performed in DMSO-d_6_, and thus the most stable conformer TN-1 was re-optimized in DMSO at the M06-2X-GD3/6-31G(d,p) theory level, while computed chemical shifts were obtained from the M06-2X/6-31++G(d,p)//DMSO method. The comparison between experimental and theoretical results is presented in Figure 3 (the total values of δ are listed in Tables S2 and S3), while the experimental NMR spectra for TN are shown in Figures S7–S10.

In a case of carmustine, a detailed discussion comparing the geometry of BCNU-1 to the theoretical molecule reported by Kamel et al. [44] and to the experimental structure [16] is provided in Text S2 (ESI). We indicate that BCNU-1 is more stable than both of these structures, and the reason for this is discussed.

As can be seen in Figure 3, the theoretical analysis is in good agreement with the experimental results. For the H-6b, H-crown (N), and H-crown (O) atoms, the theoretical δ have the same values as those obtained from the measurements. For the remaining protons, discrepancies with respect to EXP occur; however, they are rather small as the largest deviation from the experimental value is observed for the H-3 proton and amounts to 18%. It should be highlighted that some discrepancies from the measured values are rather expected, as the theoretical model is unable to fully replicate the experiment. For instance, the PCM model of the solvent used in the calculations approximately describes the solute–solvent interactions.

### 2.2. The Configurational Search, Structural, and Energetical Parameters of the TN:BCNU Complexation Process

**Figure 4:**
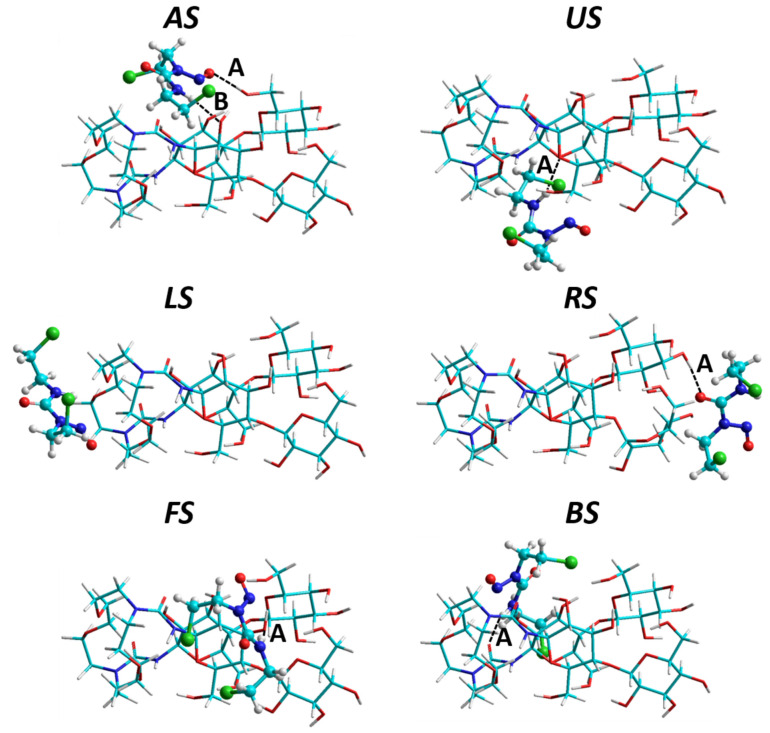
The most stable complexes selected from each configuration (Figure S1), obtained in water (PCM) at the M06-2X-GD3/6-31G(d,p) theory level. The dotted lines with letters indicate hydrogen bonds. Energetic parameters and coordinates are listed in Tables S5 and S6.

**Figure 5:**
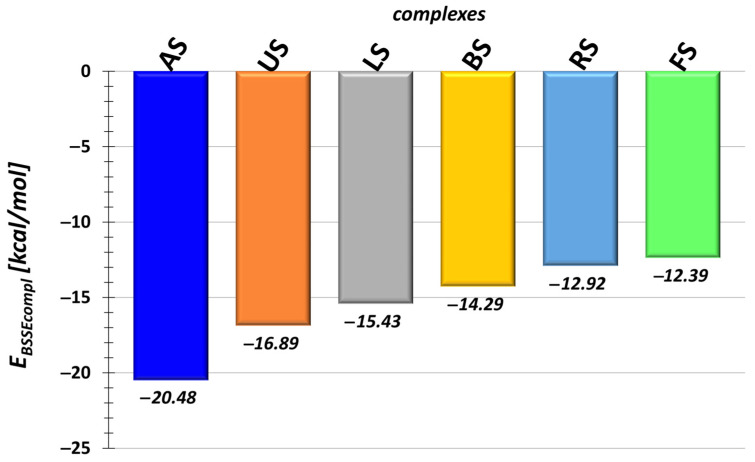
The BSSE-corrected values of complexation energy (E_BSSEcompl_) calculated for the most stable complexes (Figure 4) selected from each configuration (Figure S1).

From paper [42], we know that TN is able to form a stable complex with busulfan, which has a slightly larger molecular mass than carmustine. Thus, we expected that it will be able to form a complex with BCNU, and, indeed, this hypothesis is confirmed by both theoretical and experimental results. The most stable complexes of TN:BCNU selected from each configuration (Figure S1) and obtained in water at the M06-2X-GD3/6-31G(d,p) theory level are shown in Figure 4, while their values of the BSSE-corrected complexation energies (E_BSSEcompl_) are presented in Figure 5. In general, the results present only six out of seven configurations (see Figure S1) because the IS structure, which represents the inclusion form of complex, according to the DFT method, is not stable (value of E_BSSEcompl_ is positive). Thus, the question arises why the inclusion structure is so highly energetic. The reason for this may result from the very complex geometry of TN (see TN-1 in Figure 2). As mentioned above, we observed a large number of intramolecular hydrogen bonds that mainly form between the ureidocellobiose units. The placement of the drug between the latter may disrupt these HBs, undoubtedly increasing the energy of the carrier itself and, consequently, the complex. Thus, TN is only able to form a non-inclusion complex, in which BCNU is bound to the external part of the carrier. This is also confirmed by experiments and discussed below.

The most stable configuration is AS, in which BCNU interacts with both fragments of the carrier (the ureidocellobiosyl units and the diazacrown ether). In this structure, TN forms two HBs with BCNU, which is the largest number, as in the remaining complexes, only a single hydrogen bond is created. Again, they are moderate, and their geometrical parameters are listed in Table S4. Figure S12 presents the values of E_BSSEcompl_ for the twenty most energetically preferable structures selected from each configuration. As can be seen, the AS orientation is indeed the most favorable, as in this set, complexes from AS appear seven times.

It could be presumed that, for example, the RS orientation would be energetically favored, as it allows for direct interaction with the hydroxyl groups of the sugar units of the carrier, which should promote the formation of intermolecular hydrogen bonds. However, as observed in Figure 5, this orientation is one of the least stable. Interestingly, it even has a higher complexation energy value (~2.5 kcal/mol) than LS, where BCNU interacts only with the diazacrown ring.

In the work of Kamel et al. [44], the complexation abilities of the two graphitic carbon nitride molecules, named by the authors as CN and f-CN, towards BCNU were theoretically analyzed in water. The study was conducted using the M06-2X/6-31G(d,p) method. Admittedly, for the created complexes, they calculated not the complexation energy but the adsorption energy. Nevertheless, both energies were calculated in the same manner. According to their results, the adsorption energies are −19.05 and −22.15 kcal/mol for CN:BCNU and f-CN:BCNU, respectively. Thus, TN exhibits similar binding properties.

The complexation energy is the sum of interactions and deformations that occur during complex formation. For the most stable complexes, the energetical indicators along with the thermodynamic parameters are presented in Table 1. Analyzing the deformation energy of TN, it can be noted that this phenomenon has a somewhat favorable effect on the complexation energy, as long as it is neither too strong nor too weak. For instance, the most stable configuration, AS, is characterized by rather intermediate value of E_defTN_, while RS, one of the less stable complexes, has the highest value. A certain relationship is also evident in the case of interaction energy, as the strongest interaction is observed for the most stable complex, characterized by the highest number of hydrogen bonds, while the weakest interaction occurs in the least stable complex. As mentioned above, Table 1 also lists the thermodynamic qualities: the complexation enthalpy ($H_{\mathrm{compl}}^{\mathrm{BSSE}}$) and Gibbs energy ($G_{\mathrm{corr}\_\mathrm{compl}}^{\mathrm{BSSE}}$). Their negative values indicate that the formation of the TN:BCNU complex is exothermic and spontaneous, respectively.

Interactions between the carrier and the drug are also visible in the NMR spectrum. The comparison of the chemical shifts δ obtained for TN and BCNU in AS and in the experimental complex are listed in Table 2, along with their changes (Δδ) upon complexation. The experimental NMR spectrum obtained for the TN:BCNU complex, TN, and BCNU is presented in Figure S13.

**Figure 6:**
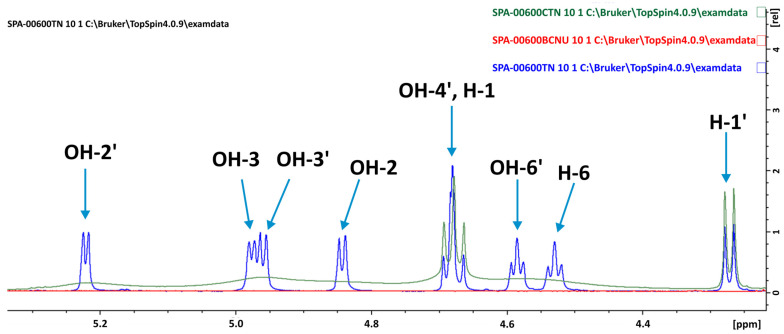
^1^H NMR spectrum obtained in DMSO-d_6_ presented for the protons belonging to the hydroxyl groups present in TN. Green line indicates the complex; blue—TN; red—BCNU.

**Figure 7:**
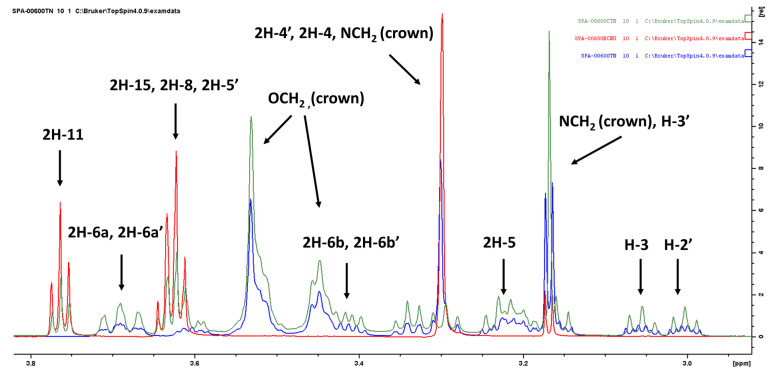
The ^1^H NMR chemical shifts obtained in DMSO-d_6_ presented for protons belonging to the diazacrown ether and the cellobiose units of TN in the free cryptand (blue line) and in the complex (green line). The red line indicates BCNU.

As can be seen in Table 2, the chemical shifts of TN in AS are in good agreement with the experimental values. The same applies to BCNU. However, as previously mentioned during the discussion of the NMR spectrum for the isolated TN, some discrepancies are rather expected, especially since the theoretical study was performed in DMSO while the measurement in DMSO-d_6_. The largest discrepancy is observed for the H-3 atom, amounting to 23% of the experimental value. In the AS complex, one of the H-3 protons (belonging to TN) is oriented towards BCNU, which undoubtedly has a strong impact on its signal value. In a case of chemical changes Δδ, the theoretical values in some cases are significantly larger and have the opposite sign. The experimental Δδ are larger for the TN protons belonging to the hydroxyl groups, the measured spectrum of which is presented in Figure 6. Thus, upon complex formation, these protons primarily interact with BCNU. This also indicates that TN forms a non-inclusion complex, which is in line with the theoretical results. Moreover, as can be seen in Figure 7, changes in the chemical shifts are observed for the protons of the diazacrown ether, proving that this fragment of TN also interacts with BCNU. It should be highlighted that this type of interaction was also observed in the theoretical model AS.

### 2.3. Cytotoxicity Assay

The toxicity of chemotherapeutic agents introduces a huge discomfort and risk to cancer patients because of the side effects. One of the most promising strategies to overcome these difficulties is the usage of carriers that could help to concentrate anticancer drugs in cancer tissues and make them less harmful for normal tissues. Drug carriers may either prolong the duration of treatments or allow increases in drug dose [45].

Cell viability was determined through the MTT assay in normal and cancer breast cells (MCF10A and MCF7, respectively), as well as normal and cancer colon cells (CCD-18Co and HT29) treated with BCNU, TN, and the TN:BCNU complex (Figure S14). The cytotoxic effect of the drug and the TN:BCNU complex was expressed as IC_50_ (Table 3), representing the drug concentration causing a 50% growth inhibition.

The results of our studies showed that carmustine exerts a cytotoxic effect against all used types of cells. Carmustine was highly cytotoxic for cells, especially breast cancer cells. The IC_50_ values for non-tumoral MCF10 and cancer MCF7 cells treated with carmustine were 282.84 ± 6.2 μM and 27.18 ± 1.4 μM, respectively. Thus, cancer cells were much more sensitive than non-tumoral cells. However, in the case of normal colon and cancer cells, the difference was not that big (63.09 ± 4.4 μM vs. 56.23 ± 3.3 μM). The cryptand alone exhibited no cytotoxicity, and the IC_50_ value was not reached. In the case of normal colon cells, it even showed a positive effect on cell viability (Figure S14). The cytotoxicity of the TN:BCNU complex was lower than that of carmustine alone in all cell types. However, IC_50_ for carmustine in a complex with the carrier was not obtained for non-tumoral MCF10A cells, but, still, the complex was effective against the MCF7 cancer cells. The reduced global toxicity of carmustine may be beneficial for therapy purposes and allow researchers to extend the usage of the drug, for example, to the treatment of feeble, overtreated, or elderly patients. The usage of carmustine in a complex with the carrier seems to be a promising strategy; however, our studies are still too preliminary to draw a final conclusion.

## 3. Materials and Methods

### Computational Details

To assess the stability of the TN:BCNU complex and characterize its structural parameters, the primary task is to find the most stable conformer for both the carrier and the drug. This is not an easy task as TN exhibits a high degree of flexibility arising from its multiple freedom levels. Therefore, a thorough exploration of its conformational surface is necessary. This was achieved by conducting a three-stage conformational analysis, in which the level of theory used gradually increased. Although carmustine is significantly smaller than the carrier and possesses few chemical groups that can rotate, a conformational search was also performed for it. A detailed description of the conformational analysis conducted for both TN and BCNU is provided in Text S1 (ESI).

For the TN:BCNU complex, a three-stage configurational search was also performed, aimed at finding the most stable complex. The strategy was very similar to that used for the isolated molecules. Briefly, the initial models of TN:BCNU were constructed in the HyperChem program [46] using the most stable conformer of TN and BCNU (Figure 2). In these structures, different orientations of drug toward TN were considered, as is presented in Figure S1, creating seven different configurations (LS; RS; AS; US; FS; BS; and IS). The new structures were generated by a systematic rotation of BCNU around each axis X, Y, and Z, gradually varying the angle by 20°. This method allowed for obtaining 40,830 complexes, which were first optimized in vacuo using AMBER99 [47,48] and further reoptimized at the PM7 level of theory [49] in the MOPAC16 program [50]. This selection was based on previous studies showing the successful application of these methods to molecules with geometries similar to TN [51,52,53]. Also, the results of the conformational search performed for TN (Text S1) indicate that the most stable conformers obtained from the M06-2X-GD3/6-31G(d,p) calculations (a description of this method is given below) are from the AMBER99 set. From the PM7-optimized set, 105 different structures (15 from each configuration) were selected for the final step of the configurational analysis performed at the density functional theory (DFT) level. The optimizations were performed using the meta exchange correlation functional (M06-2X) [54] with the Pople basis set 6-31G(d,p) [55]. During the calculations, the dispersion interactions were described by the Grimme empirical dispersion corrections (GD3) [56], while the presence of solvent water was described by the polarizable continuum model of solvent PCM. The rationale for choosing this method is explained in Text S1 (ESI). The DFT calculations were performed in the Gaussian16 program (Revision C.01) [57]. The vibrational frequency calculations were carried out at the same theory level as the optimizations to obtain the thermodynamic parameters such as enthalpy (H) and Gibbs energies (G), as well as to confirm that the optimized complexes are true minima on the potential energy surface. The GoodVibes v2.0.3 program [58,59] was employed to recalculate the Gibbs energy values by including quasiharmonic free energy corrections for the low vibrational frequencies. The corrected Gibbs energy values are labeled as G_corr_.

The complexation and interaction energies were calculated using the supermolecular approach according to the equations given below:

(1) $$E_{compl}=E_{complex}^{OPT}-\left(E_{TN}^{OPT}+E_{BCNU}^{OPT}\right)$$

(2) $$E_{int}=E_{complex}^{OPT}-\left(E_{TN}^{SP}+E_{BCNU}^{SP}\right)$$

where $E_{complex}^{OPT}$ is the energy of the optimized complex; $E_{TN}^{OPT}$ and $E_{BCNU}^{OPT}$ are the energies of TN and BCNU in their most stable geometries; and $E_{TN}^{SP}$ and $E_{BCNU}^{SP}$ are the single-point energies of the host TN and BCNU, respectively, taken from the PCM-optimized complex.

## 4. Experimental Details

### 4.1. Synthesis of TN

**Scheme 1:**
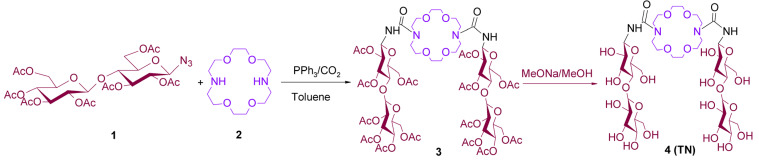
Schematic procedure of synthesis **4** (TN).

The bis-cellobiosyl-diazacrown ligand **4** (TN) was synthesized in two steps according to the mechanism described in reference [42] and schematically shown in Scheme 1. Briefly, TN was synthesized via the Staudinger–aza-Wittig reaction [60,61] (also known as the phosphine imide reaction) [62,63], in which β-ᴅ-cellobiosyl azide **1** and diazacrown ether **2** were mixed to give the acetylated form of TN **3** with a high yield (99%). Zemplén conditions were used to deacetylate **3**, finally yielding TN **4**. The proposed geometry of the latter was confirmed by means of spectroscopic measurements (^1^H NMR and COSY), the details of which are provided in Text S3 (ESI).

### 4.2. Synthesis of the TN:BCNU Complex

Carmustine and TN were mixed in a 1:1 molar ratio in deuterated DMSO for 24 h, after which the ^1^H NMR spectrum was measured. Overlaying the ^1^H NMR spectra of carmustine, TN, and the TN:BCNU complex revealed changes in the shifts of some protons (Figure S13), indicating that the complex had formed.

### 4.3. MTT Assay

Human cell lines MCF10A (CRL-10317), MCF7 (HTB-22), HT29 (HTB-38), and CCD-18Co (CRL-1459) were obtained from the American Type Culture Collection (Manassas, VA, USA).

Breast cancer cells MCF7, colon cancer cells HT29, normal breast epithelial cells MCF10A, and normal colon fibroblasts CCD-18Co were used as a cell model for testing the effect of compounds on the viability of cells. Cells were seeded in Dulbecco’s Modified Eagle Medium (DMEM) in 96-well plates and incubated for 24 h in a 5% CO_2_ atmosphere at 37 °C. MCF10A cells were grown in a medium supplemented with 0.4% bovine pituitary extract (BPE), 3 ng/mL hEGF, 5 μg/mL insulin, 0.5 μg/mL hydrocortisone, and 100 ng/mL cholera toxin. After that, the cells were treated with different concentrations—2.5, 10, 25, 50, 100, 200, and 400 µM—of compounds for 24 h. The viability of the cells treated with the tested compounds was assessed by measuring the ability of live cells to metabolize 3-(4,5-dimethylthiazolo-2-yl)-2,5-diphenyl tetrazolium bromide (MTT) to formazan, using a standard protocol. Briefly, after incubation with compounds, the medium of each well was discarded, the cells were washed with PBS, and then 100 μL of fresh media with 10 μL MTT solution (5 mg mL − 1 in PBS) was added to each well. After 4 h incubation at 37 °C, the produced formazan was solubilized through the addition of DMSO, and the absorbance of each well was determined at 570 nm using an ELISA reader. The results were expressed as the mean of at least four replicates as a percentage of control (taken as 100%—cells).

## 5. Conclusions

In this work, we conducted a combined experimental and theoretical study of the molecule 1,10-*N*,*N’*-bis-(β-ᴅ-ureidocellobiosyl)-4,7,13,16-tetraoxa-1,10-diazacyclooctadecane (TN), which can be considered as a potential drug carrier for various anticancer drugs. For this purpose, we chose the small and cytotoxic anticancer drug carmustine. Detailed theoretical analysis allowed us to identify the most energetically favorable geometries of all molecules: TN, carmustine, and their complex. TN is characterized by a very compact geometry due to a large number of intramolecular hydrogen bonds, primarily formed between the hydroxyl groups of the ureidocellobiosyl units. This compact geometry significantly impacts its complexation abilities, as TN can only form non-inclusion complexes, in which carmustine is bound to the cellobiose units via intermolecular hydrogen bonds. The stability of this complex is estimated to be around −20.5 kcal/mol. The non-inclusion geometry of the complex is also confirmed by experimental measurements performed in DMSO-d_6_.

Cytotoxicity studies were conducted on all molecules using two normal and two cancer cells: breast and colon. As expected, carmustine was highly cytotoxic; however, when bound to TN, its cytotoxicity was reduced, which had a positive effect on normal cells. Additionally, TN alone was not cytotoxic.

Therefore, the non-toxicity of TN, as well as its ability to form stable complexes with small and toxic anticancer drugs, suggests that TN can be considered as a potential drug carrier. Furthermore, it is worth verifying its complexation abilities with larger drugs, and we are planning to conduct such studies in the future.

## Figures and Tables

**Table 1:** The BSSE-corrected interaction energies ($E_{\mathrm{INT}}^{\mathrm{BSSE}}$), complexation enthalpies ($H_{\mathrm{compl}}^{\mathrm{BSSE}}$), and complexation Gibbs energies ($G_{\mathrm{corr}\_\mathrm{compl}}^{\mathrm{BSSE}}$) as well as the deformation energies (*E_def_*) for the most stable TN:BCNU complexes presented in Figure 4. All values are in kcal/mol.

Molecule	$E_{INT}^{BSSE}$	*E_defTN_*	*E_defBCNU_*	*E_tot_*	$G_{corr\_compl}^{BSSE}$	$H_{compl}^{BSSE}$
***AS***	−27.26	1.86	4.92	6.78	−8.98	−18.75
***US***	−21.25	1.56	2.80	4.36	−5.88	−15.47
***LS***	−17.98	0.48	2.08	2.55	−5.14	−14.10
***BS***	−17.43	0.53	2.61	3.14	−4.07	−12.76
***RS***	−21.50	7.08	1.50	8.58	−0.92	−10.70
***FS***	−15.25	0.55	2.32	2.86	−3.18	−11.33

**Table 2:** The values of the ^1^H scaled chemical shifts (δ) [ppm] of TN and BCNU obtained from the theoretical (δ_CAL_) and experimental (δ_EXP_) complex, as well as their changes Δδ calculated as Δδ = δ_TN/BCNU in complex_ − δ_TN/BCNU isolated_.

TN in Complex					BCNU in Complex				
Number of Atoms (Figure 1)	δ_CAL_ of TN in AS	δ_EXP_ of TN in Complex	Δδ_CAL_	Δδ_EXP_	Number of Atoms (Figure 1)	δ_CAL_ of BCNU in AS	δ_EXP_ of BCNU in Complex	Δδ_CAL_	Δδ_EXP_
H-1	5.11	4.68	0.17	−0.002	NH	7.67	8.96	0.63	−0.0012
H-2	3.14	3.23	−0.02	−0.0014	H-8	3.87	3.62	−0.09	−0.001
H-3	3.75	3.06	0.13	−0.0005	H-11	3.71	3.76	0.34	−0.0008
H-4	3.57	3.29	−0.05	−0.0006	H-15	3.21	3.76	−0.27	0.1295
H-5	3.62	3.23	0.03	0.035	H-18	3.99	4.10	0.33	0.0008
H-6a	4.04	3.69	0.02	−0.0121					
H-6b	3.41	3.44	−0.05	−0.0029					
H-1′	4.40	4.27	0.01	−0.0001					
H-2′	3.32	3.06	0.02	0.0525					
H-3′	3.41	3.16	0.09	−0.0042					
H-4′	3.07	3.34	0.06	0.0075					
H-5′	3.67	3.23	0.14	−0.001					
H-6a’	3.76	3.69	0.00	0.0119					
H-6b’	3.50	3.60	0.02	0.0002					

**Table 3:** The cytotoxicity assay (IC_50_ values (μM)) performed for the analyzed molecules: carmustine (BCNU) and the TN:BCNU complex.

Cell Lines	Carmustine	TN:BCNU
CCD-Co18	63.09 ± 4.4	199.52 ± 5.9
HT29	56.23 ± 3.3	158.49 ± 7.8
MCF10A	282.84 ± 6.2	-
MCF7	27.18 ± 1.4	89.12 ± 2.7

## Data Availability

The data presented in this study are available in article and Supplementary Materials.

## Supplementary Materials

The following supporting information can be downloaded at: https://www.mdpi.com/article/10.3390/molecules29143359/s1, Text S1: Description of the conformational analysis performer for TN and BCNU; Figure S1: Graphical representation of initial model of complexes; Figure S2: Torsion angles marked in TN; Figure S3: Torsion angles marked in BCNU; Figure S4: Less stable conformers of TN; Figure S5: Less stable conformers of BCNU; Table S1: Geometrical parameters of HBs formed in TN; Text S2: Comparison of theoretical results with the literature data for BCNU; Figure S6: Relative energy differences obtained from various DFT methods for TN and BCNU; Text S3: Experimental details concerning TN synthesis and spectroscopic details; Figure S7: Experimental 1H NMR spectrum of TN; Figure S8: Experimental 1H NMR spectrum of the OH protons of TN; Figure S9: Experimental 1H NMR spectrum of the diazacrown ether protons of TN; Figure S10: COSY spectrum of TN; Figure S11: Experimental 1H NMR spectrum of BCNU; Table S2: The 1H NMR chemical shifts of BCNU-1 obtained from calculations; Table S3: The 1H NMR chemical shifts of TN-1 obtained from calculations; Figure S12: The BSSE-corrected complexation energies for the 20 TN:BCNU complexes; Table S4: The geometrical parameters of HBs formed in the most stable TN:BCNU complexes; Table S5: The energetical parameters of the most stable TN:BCNU complexes; Table S6: The coordinates of the most stable TN:BCNU complexes; Figure S13: Overlapping 1H NMR spectra of complex, BCNU, and TN; Figure S14: Effect of BCNU, TN, and TN:BCNU complex on normal and cancer cells. References [16,42,44,46,47,48,49,50,51,52,53,54,55,56,57,58,59,64,65,66,67,68,69,70] are cited in the Supplementary Materials.
