# Supplementary material for: Combined Theoretical and Experimental Investigations: Design, Synthesis, Characterization, and In Vitro Cytotoxic Activity Assessment of a Complex of a Novel Ureacellobiose Drug Carrier with the Anticancer Drug Carmustine
Source: Molecules. 2024 Jul 17;29(14):3359. doi: 10.3390/molecules29143359 (PMC11280079; doi:10.3390/molecules29143359)
Supplement: Supplementary file 1 [file molecules-29-03359-s001.zip › molecules-3048990-supplementary.pdf]

Electronic Supplementary File (ESI)

Combined theoretical and experimental investigations: design, synthesis, characterization, and in vitro cytotoxic activity assessment of a complex of a novel ureacellobiose drug carrier with the anticancer drug carmustine

Marta Hoelm[a*], Stanisław Porwański[b], Paweł Jóźwiak[c] and Anna Krześlak[c]

[a] Theoretical and Structural Group, Department of Physical Chemistry, Faculty of Chemistry, University of Lodz, Pomorska 163/165, 90-236 Lodz, Poland

[b] Department of Organic and Applied Chemistry, Faculty of Chemistry, University of Lodz, Tamka 12, 91-403 Lodz, Poland

[c] Department of Cytobiochemistry, Faculty of Biology, University of Lodz, Pomorska 141/143, 90-236 Lodz, Poland

* Corresponding author. E-mail: marta.hoelm@chemia.uni.lodz.pl

Table of Contest

Text S1: Description of the conformational analysis performer for TN and BCNU....................3

Figure S1: Graphical representation of initial model of complexes...4

Figure S2: Torsion angles marked in TN..5

Figure S3: Torsion angles marked in BCNU..6

Figure S4: Less stable conformers of TN 7

Figure S5: Less stable conformers of BCNU 8

Table S1: Geometrical parameters of HB formed in TN 8

Text S2: Comparison of theoretical results with the literature data for BCNU 9

Figure S6: Relative energy differences obtained from various DFT methods for TN and BCNU 9

Text S3: Experimental details concerning the TN synthesis and spectroscopic details............10

Figure S7: Experimental ^{1}H NMR spectrum of TN 11

Figure S8: Experimental ^{1}H NMR spectrum of the OH protons of TN 11

Figure S9: Experimental ^{1}H NMR spectrum of the diazacrown ether protons of TN 12

Figure S10: COSY spectrum of TN 12

Figure S11: Experimental ^{1}H NMR spectrum of BCNU 13

Table S2: The ^{1}H NMR chemical shifts of BCNU-1 obtained from calculations........................13

Table S3: The ^{1}H NMR chemical shifts of TN-1 obtained from calculations 13

Figure S12: The BSSE corrected complexation energies for the 20 TN:BCNU complexes 15

Table S4: The geometrical parameters of HB formed in the most stable TN:BCNU complexes 15

Table S5: The energetical parameters of the most stable TN:BCNU complexes 16

Table S6: The coordinates of the most stable TN:BCNU complexes 16

Figure S13: Overlapping ^{1}H NMR spectra of complex, BCNU and TN.....................................23

Figure S14: Effect of BCNU, TN and TN:BCNU complex on normal and cancer cells.............23

References: 24

Text S1: The description of the conformational analysis performed for the drug carrier and carmustine.

The initial models of TN and BCNU were built using the HyperChem program [1]. For TN, we used its acetylated form obtained from X-ray study [2] and then replaced the acetyl groups with hydrogen atoms. For BCNU, the coordinates were obtained from the DrugBank database (DrugBank Accession Number DB00262) [3]. The experimental coordinates of BCNU reported in [4] were not used, as we aimed to validate our conformational search strategy for small compounds like carmustine. The results are compared to experimental data provided in Text S2 below.

The conformational search performed for both TN and BCNU closely followed the methods used in our previous work [5–7]. The first stage was related with the calculations performed in vacuo at the molecular mechanics theory level using the Conformational Search module available in HyperChem. During this analysis, new conformations were generated by random changes of 14 torsion angles marked in TN (Fig. S2) and 9 in carmustine (Fig. S3). As a result, 211800 and 765 structures of TN and BCNU, respectively, were obtained. All these conformers were optimized using three different force fields available in HyperChem: MM+ [8]; AMBER99 [9,10] and CHARMM27 [11]. The potential energy surface of carmustine is significantly less corrugated; thus, its conformers were optimized using only AMBER99.

In the second stage, all structures were reoptimized in vacuo using the semiempirical method PM7 [12] and the MOPAC2016 program [13]. Three structures indicated as the most stable were used as starting points in the Stochastic Dynamic simulations with the Verlet algorithm [14]. Each simulation was conducted at T=500K (the high temperature allows for overcoming the energy barriers which may occur on the potential energy surface) during the period of 20 ps long with a time-step 10 fs. From the trajectory, the 100 lowest energy structures were selected and reoptimized at the PM7 level. The calculations were performed in the Gabedit program [15] combined with MOPAC 2016. Molecules obtained from the trajectory were combined with the semiempirical results and ranked according to the increasing heat of formation (ΔH_f).

The final step of the conformational analysis was conducted at the density functional theory (DFT) level for the 400 and 700 conformers of carrier and carmustine, respectively, selected from the second stage. Calculations were performed using the Gaussian16 program

[15] and the M06-2X-GD3/6-31G(d,p) method, which includes the hybrid meta-GGA exchange-correlation functional M06-2X with the Grimme GD3 empirical corrections [16,17] and the Pople's 6-31G(d,p) basis set. [18]. The choice of this method was dictated by its good performance for the thermochemistry and description of the noncovalent interactions [16,19]. The prediction of the M06-2X-GD3 configuration ranking was further verified by performing optimizations (BCNU) or single point calculations (TN) using the same functional, but larger basis sets: 6-31++G(d,p) and 6-311++G(d,p). The vibrational analysis was performed at the M06-2X-GD3/6-31G(d,p) theory level to confirm that the optimized structures correspond to true minima and to obtain the thermochemistry quantities. One of them, the Gibbs energy, was further recalculated in the GoodVibes program [20,21] and denoted as G_{corr}. All calculations performed at the DFT theory level were conducted with the presence of solvent (water) described by the polarizable continuum model PCM.

The NMR calculations were performed using the Gauge-Independent Atomic Orbital (GIAO) approach [22]. The NMR shielding for the hydrogen and carbon atoms was calculated in DMSO (PCM) at the M06-2X/6-31++G(d,p) theory level. The chemical shifts δ were received using the approach proposed by Tantillo [23], where they are computed according to the formula $\delta = (I-\sigma)/(-S)$, with σ being the isotropic value obtained from the DFT calculations, and I and S being the scaling factors. The mean scaled δ values were determined by calculating the arithmetic average of the chemical shifts of atoms with identical numbers in both cellobiosyl units.

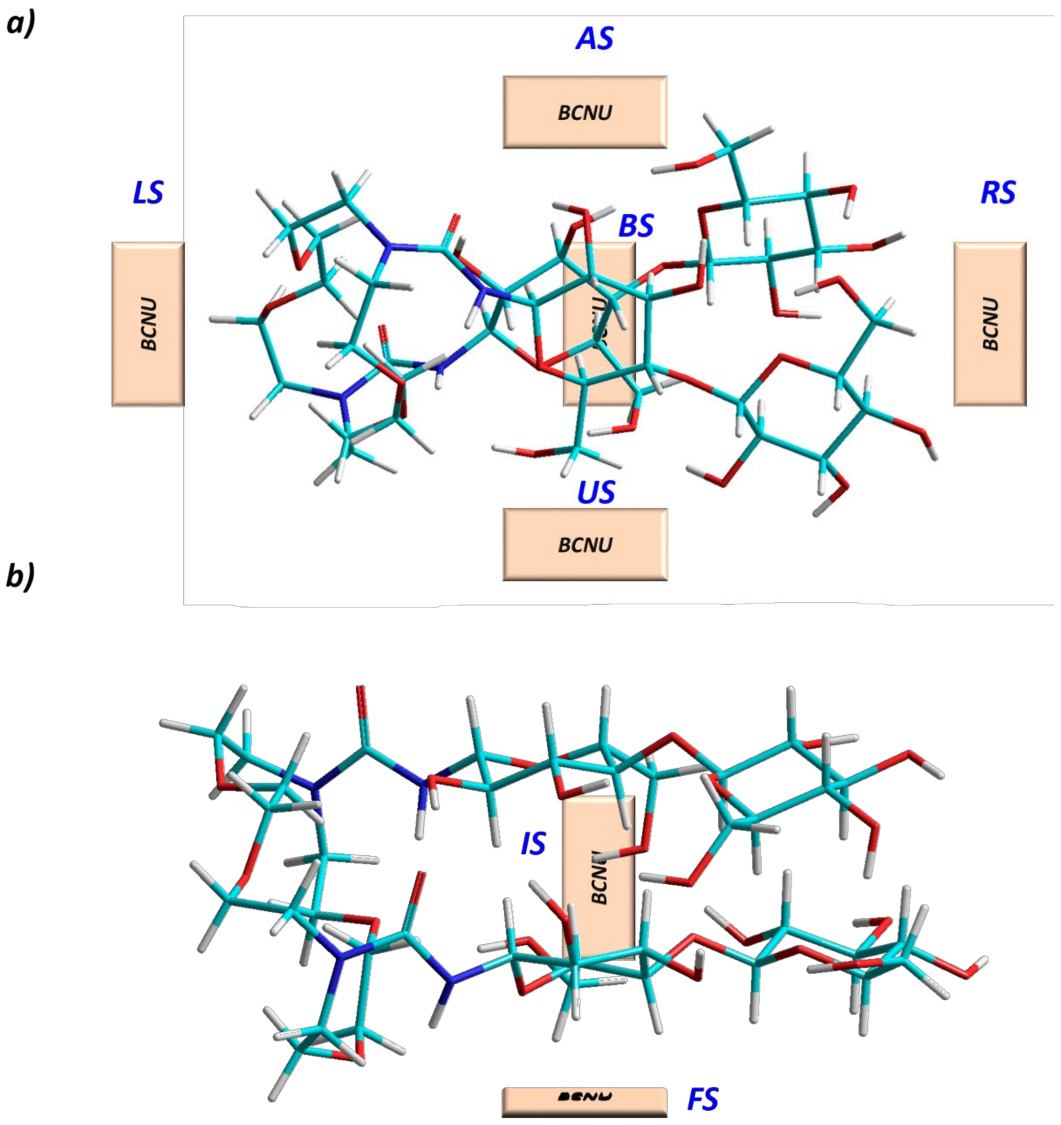

Figure S1. The graphical representation of the seven orientation (**LS; RS; AS; US; FS; BS; IS)** of carmustine (**BCNU; presented as rectangle**) towards 1,10-*N*,*N′*-bis-(β-D-ureidocellobiosyl)-4,7,13,16-tetraoxa-1,10-diazacyclooctadecane (TN) (a). For clarity in view (b), TN is rotated by 90° about X axis.

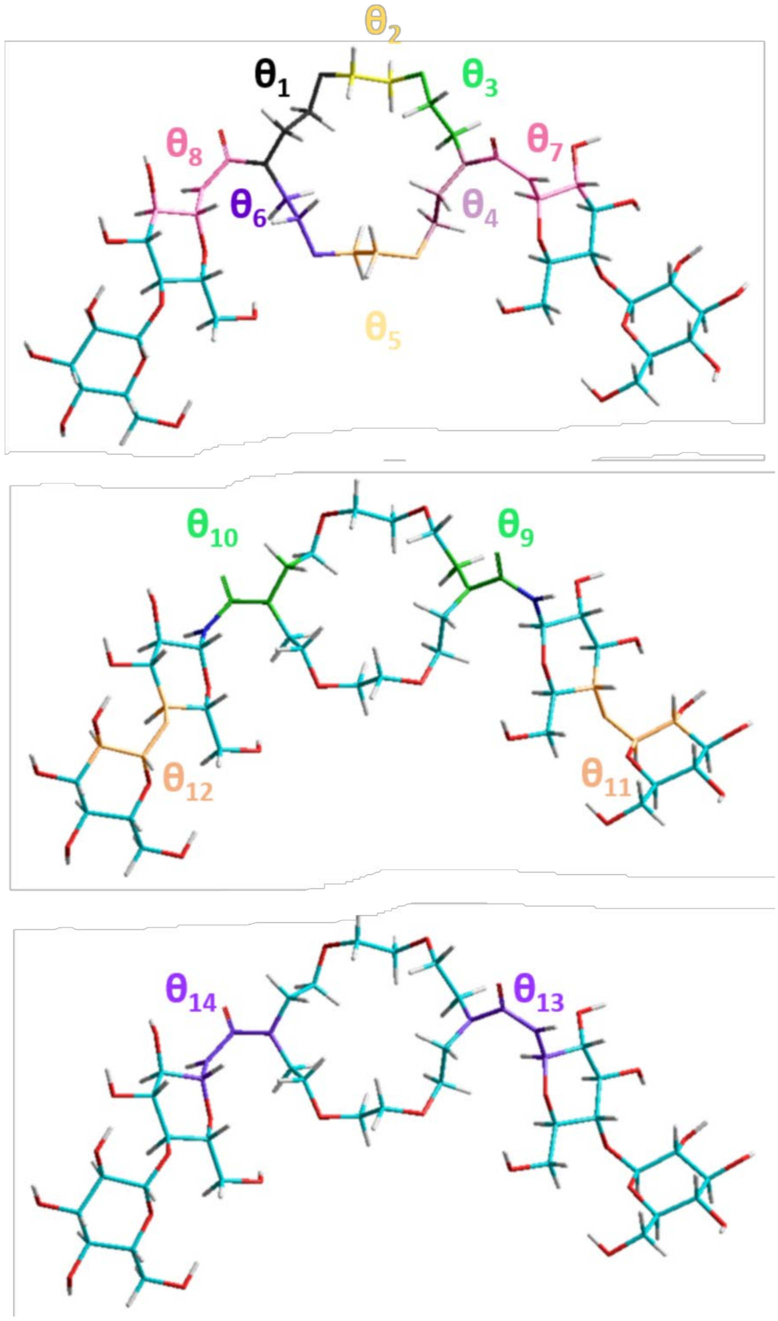

Figure S2. The torsion angles (θ) marked in TN, and changed during the conformational search performed in the HyperChem program.

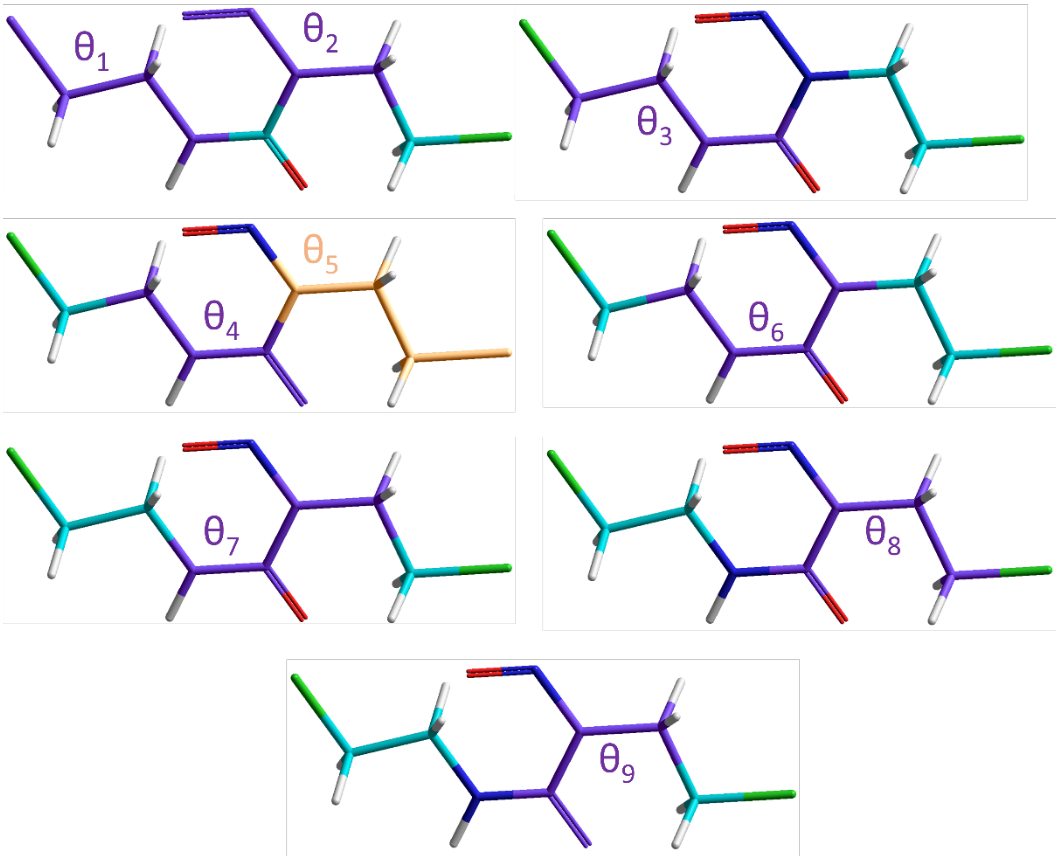

Figure S3. The torsion angles (θ) marked in carmustine, and changed during the conformational search performed in the HyperChem program.

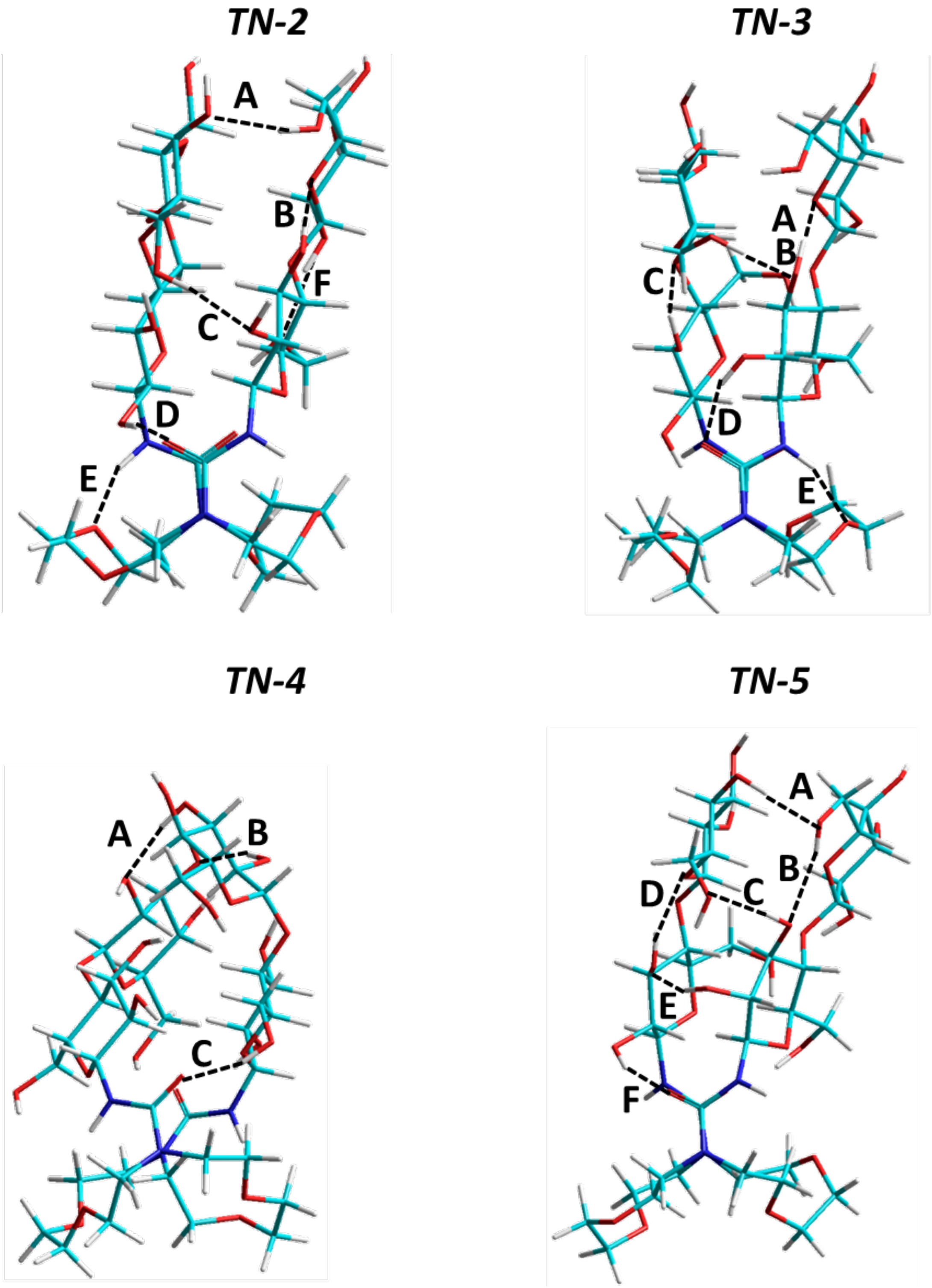

Figure S4. Less energetically preferable conformers of TN. The dotted lines with the letters indicate hydrogen bonds, which geometrical parameters are listed in Table S1.

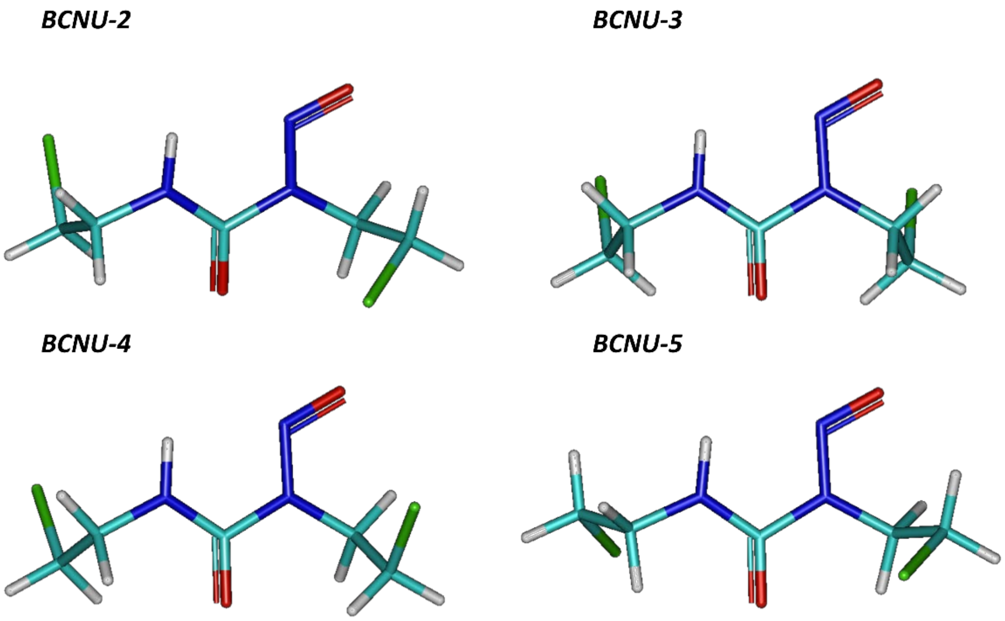

Figure S5. Less energetically preferable conformers of carmustine (BCNU).

Table S1. The geometrical parameters of hydrogen bonds formed in the most stable conformers of TN.

Conf.	Symb.	bond O/N-H···O	$d_{O/N-H}$ [Å]	$d_{H···O}$ [Å]	$d_{O···O}$ [Å]	∠ O/N-H···O [°]
**TN-1**	A	O30-H42···O72	0.978	1.854	2.823	169.6
	B	O72-H84···O93	0.976	2.225	3.135	154.6
	C	O93-H102···O29	0.979	1.825	2.761	159.0
	D	O50-H59···O25	0.974	1.990	2.893	153.3
	E	O70-H81···O53	0.979	1.757	2.726	169.9
	F	O96-H106···O6	0.970	2.172	3.083	155.8
	G	N131-H135···O3	1.071	1.950	2.827	142.8
**TN-2**	A	O72-H84···O30	0.972	1.911	2.848	160.9
	B	O93-H102···O68	0.975	1.971	2.874	152.9
	C	O29-H41···O94	0.972	1.962	2.926	170.7
	D	O51-H60···O134	0.976	1.795	2.762	170.7
	E	N132-H136···O114	1.019	1.897	2.842	153.0
	F	O70-H81···O96	0.978	1.918	2.885	169.3
**TN-3**	A	O93-H102···O68	0.977	1.872	2.788	154.9
	B	O29-H41···O93	0.976	1.927	2.836	153.8
	C	O50-H59···O25	0.973	1.850	2.770	156.4
	D	O94-H103···O134	0.977	1.868	2.800	158.6
	E	N131-H135···O3	1.021	1.875	2.850	158.5
**TN-4**	A	O31-H43···O70	0.978	1.832	2.798	168.6
	B	O27-H38···O74	0.975	1.916	2.850	159.8
	C	O53-H63···O134	0.970	1.809	2.727	156.6
**TN-5**	A	O30-H42···O72	0.980	1.853	2.822	169.7
	B	O72-H84···O93	0.976	2.274	3.186	154.9
	C	O93-H102···O29	0.979	1.810	2.749	159.8
	D	O50-H59···O25	0.974	1.897	2.806	154.1
	E	O94-H103···O50	0.977	1.973	2.883	154.1
	F	O51-H60···O134	0.970	1.860	2.773	155.6

Text S2. The comparison of theoretical results obtained for carmustine with literature data.

The theoretical analysis aimed at finding the most stable structure of carmustine was performed by Kamel et al. [24]. Their most energetically preferable structure, denoted as **BCN**, is presented in Figure 1 in [24]. During our conformational search, BCN was also received; however, its energy is 0.8 kcal/mol higher than that of BCNU-1 (see Fig. 2 in the main article). Such a small energy difference results from the rotation about 90° one of the chloromethane group present in BCNU-1. From the Cambridge Structural Database (CCDC), we took the coordinates of the BCNU crystal structure [4], which were optimized at the M06-2X-GD3/6-31G(d,p) theory level in water (PCM). The energy of the optimized structure is also 0.35 kcal/mol higher than that of BCNU-1. Therefore, to the configurational search of the TN:BCNU complex, BCNU-1 (presented in Figure 2 in the main article) was chosen.

a) TN

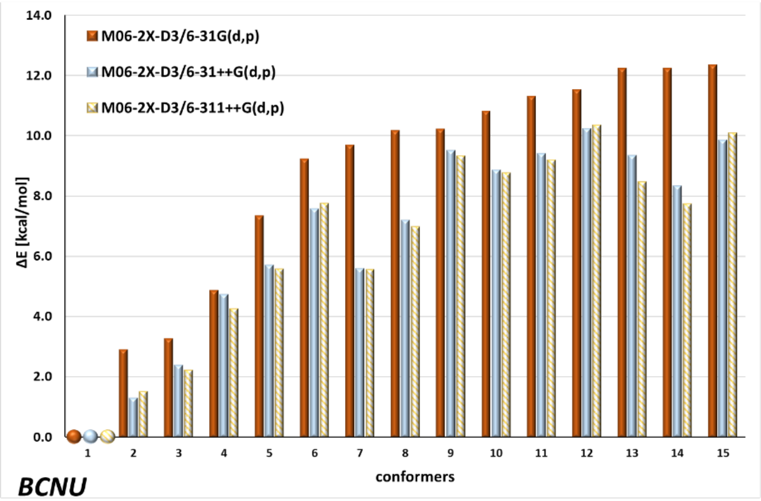

b) BCNU

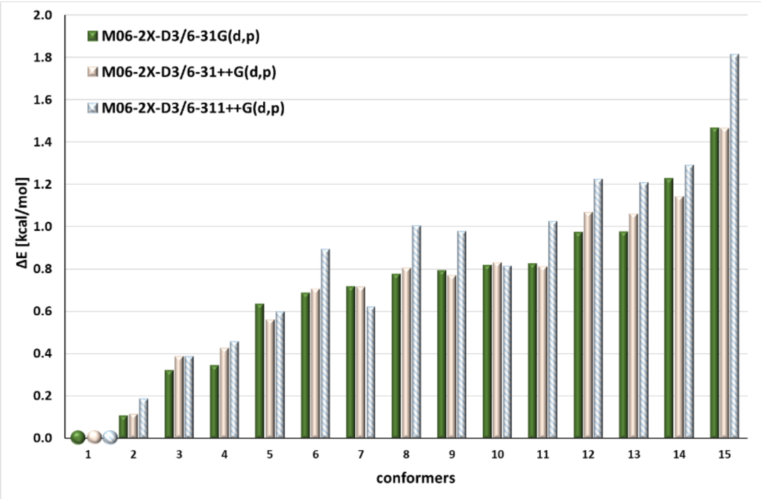

Figure S6. The relative energy differences ΔE [kcal/mol] obtained from the various DFT methods, calculated as a difference between the most stable conformer (1) and other remaining (from 2 to fifteen). For carmustine (b), optimizations were performed, while for TN (a), optimizations were conducted only with the small 6-31G(d,p) basis set; for the rest (6-31++G(d,p) and 6-311++G(d,p)), single point calculations were done.

Text S3. The experimental details concerning the synthesis and spectroscopic analysis of **TN** and its acetylated form (**ATN**).

All reagents and solvents were purchased from Merck and used as supplied. NMR spectra were recorded in $CDCl_3$ and DMSO-d_6, on Bruker Avance III (600MHz for ^1H NMR, 150MHz for ^{13}C NMR), coupling constants are reported in Hz. The progress of the reactions was monitored by silica gel thin-layer chromatography plates (Merck TLCSilicagel60 F254).

*Synthesis of 1,10-N,N′-bis-(2,3,6,2′,3′,4′,6′-hepta-O-acetyl-β-D-ureidocellobiosyl)-4,7,13,16-tetraoxa-1,10-diazacyclooctadecane (**ATN**).*

A solution (661mg, 1mmol) of 2,3,6,2′,3′,4′,6′-hepta-O-acetyl-azido-β-D-cellobiose and triphenylphosphine (3mmol) in anhydrous toluene (20 ml) was stirred for 1h at rt, then 4,7,13,16-tetraoxa-1,10-diazacyclooctadecane (130mg, 0.5mmol) was added to the mixture and stirred for 24 h under CO_2 bubbling. After evaporation of the toluene, the residue was chromatographed on silica gel column (eluent AcOEt/MeOH, 8/1).

*Synthesis of 1,10-N,N′-bis-(β-D-ureidocellobiosyl)-4,7,13,16-tetraoxa-1,10-diazacyclooctadecane (**TN**).*

In a solution of MeOH/MeONa (50ml) the compound ATN (0.5mmol) was added. The resulting mixture was stirred at room temperature for 24 h and was filtered through a short column of resin Dovex 50WX8 hydrogen form. The filtrate was evaporated and the TN was obtained as a white powder with quantitative yield and used without purification.

NMR ^{1}H (600MHz, DMSO); 6.79(d, 2H, 2NH, J=8.5); 5.22(d, 2H, 2OH, 2OH-2′, J=4.6); 4.98(d, 2H, 2OH, 2OH-3, J=4.8); 4.96(d, 2H, 2OH, 2OH-3′, J=5.2); 4.84(d, 2H, 2OH, 2OH-2, J=5.6); 4.69(d, 2H, 2OH, 2OH-4′, J=6.2); 4.68(t, 2H, 2H-1, J=9.6, 8.0); 4.58(t, 2H, 2OH, 2OH-6′, J=5.2); 4.53(t, 2H, 2OH, 2OH-6, J=5.6); 4.27(d, 2H, 2H-1′, J=7.9); 4.10-4.04(m, 2H, 2H-4):3.74-3.67(m, 2H, 2H-6a);3.68-3.65(m, 2H,2H6′a); 3.64-3.60(m, 2H, 2H-5′); 3.56-3.43(m, 8H, H-crown); 3.46-3.39(m, 2H, 2Hb); 3.43-3.38(m, 2H, 2H6′b); 3.37-3.32(m, 2H, 2H-4′); 3.32-3.28(m, 8H, H-crown); 3.32-3.27(m, 2H, 2H-4); 3.27-3.20(m, 2H, 2H-2); 3.25-3.19(m, 2H, 2H-5); 3.18-3.16(m, 8H, H-crown); 3.20-3.14(m, 2H, 2H-3′); 3.08-3.03(m, 2H, 2H-3); 3.03-2.98(m, 2H, 2H-2′)

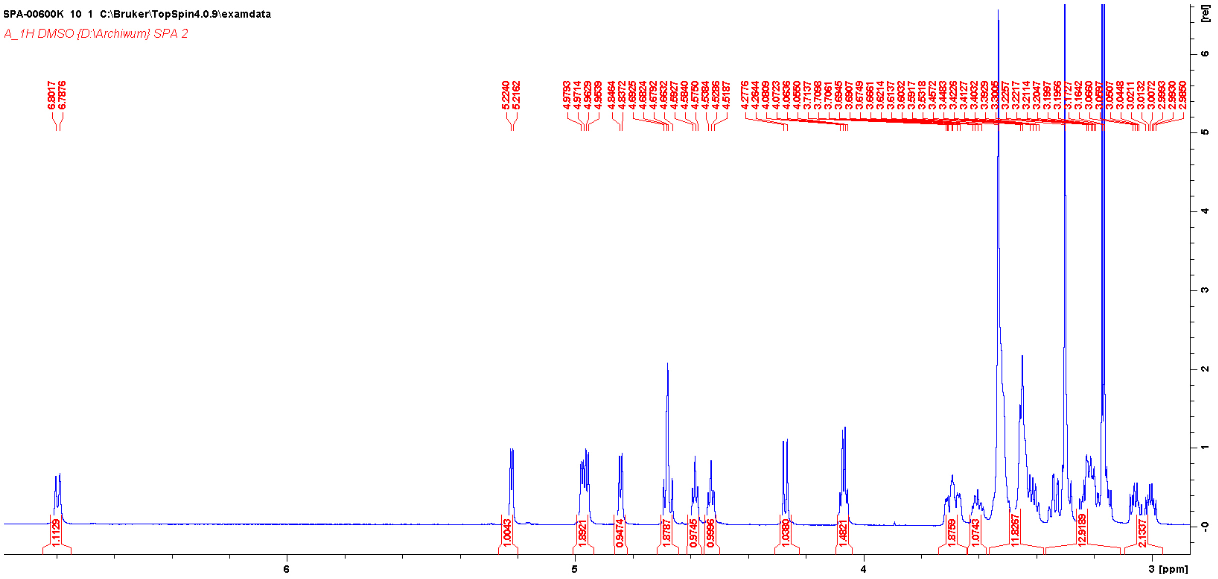

Figure S7. Experimental ^1H NMR spectrum of TN obtained in DMSO-d_6.

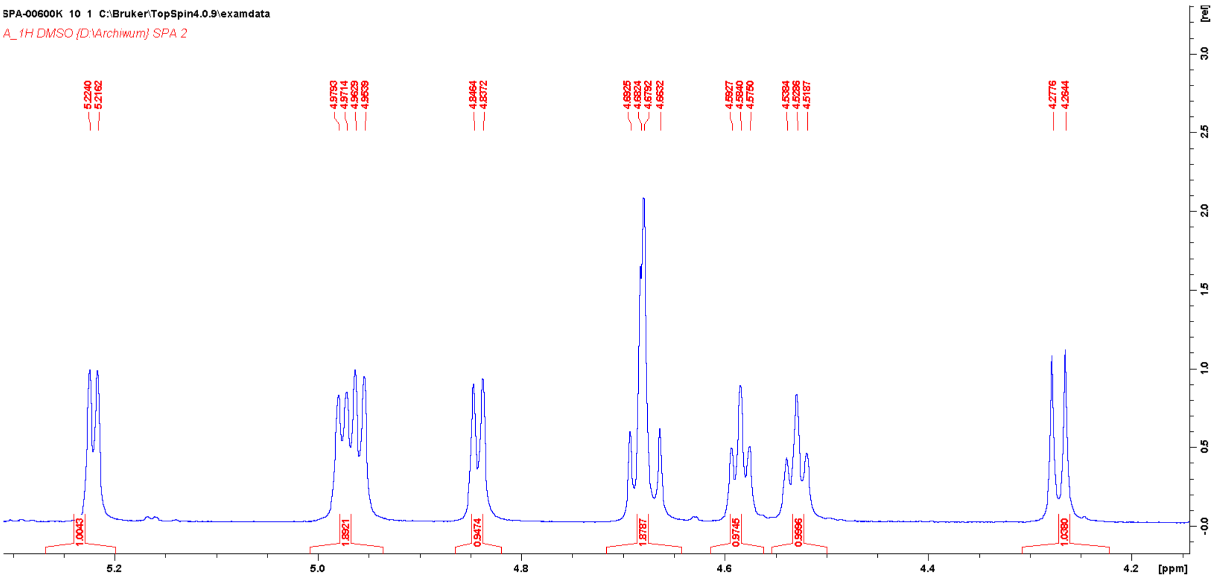

Figure S8. Experimental ^1H NMR spectrum of the protons from the hydroxyl groups of TN obtained in DMSO-d_6.

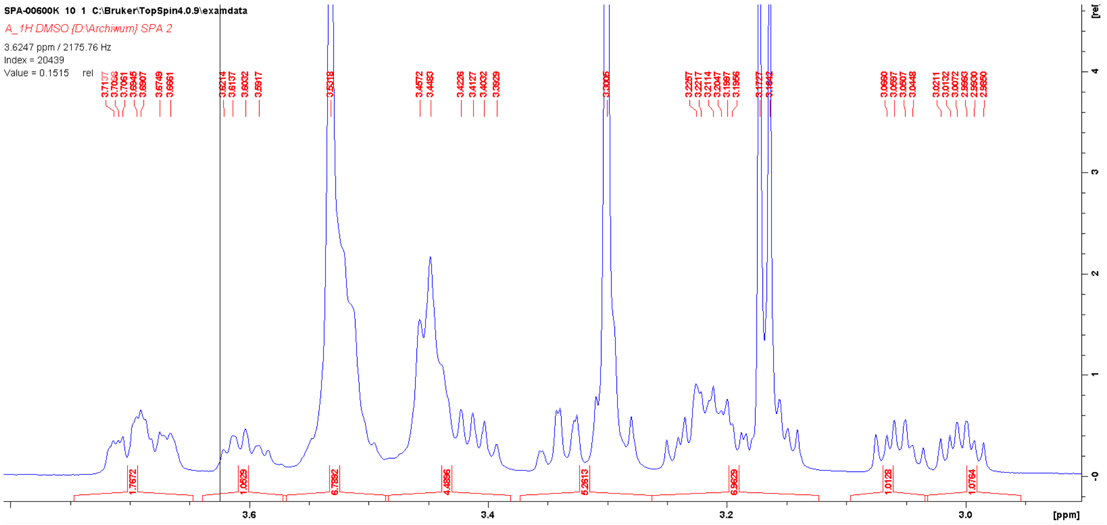

Figure S9. ^{1}H NMR chemical shifts of the diazacrown ether protons of TN obtained in DMSO-d6.

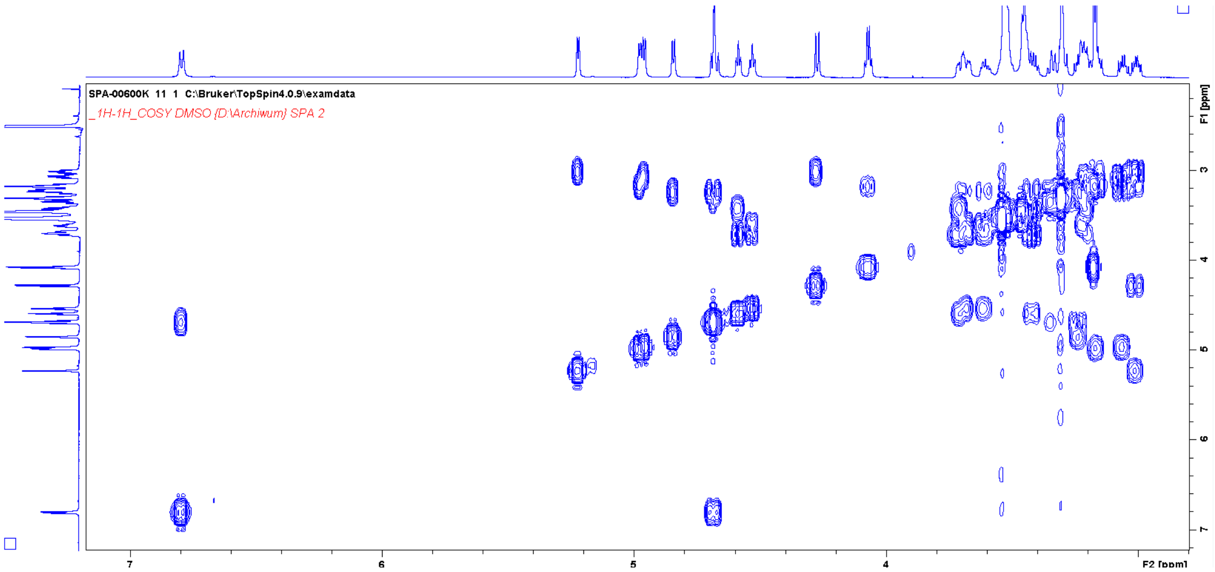

Figure S10. The COSY spectrum of TN

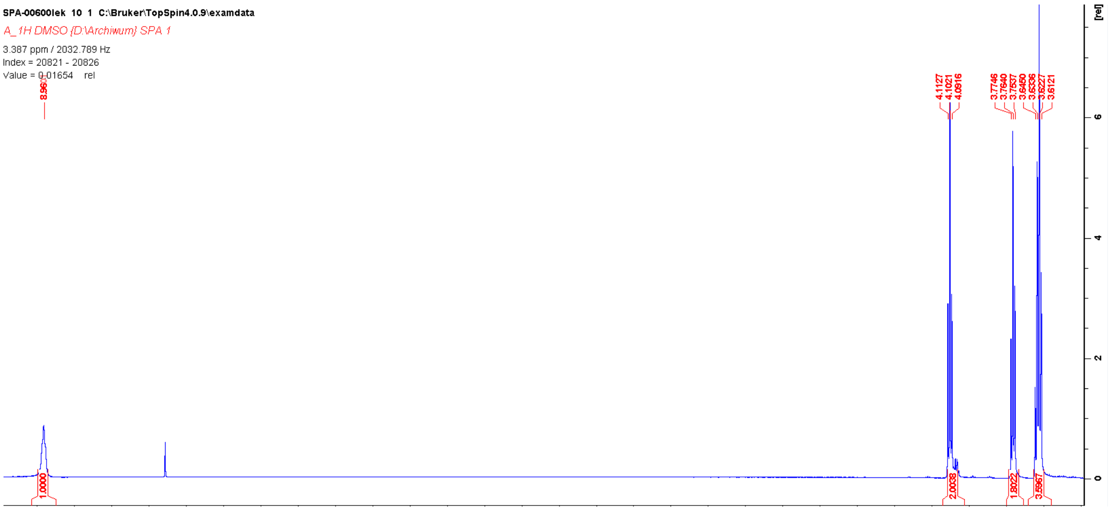

Figure S11. ^{1}H NMR spectrum of carmustine.

Table S2. The ^{1}H NMR chemical shifts (scaled) of the isolated carmustine (Fig. 2 in the main article) obtained from the M06-2X/6-31++G(d,p) calculations in DMSO.

<table>
<tr><th>Atom numbers (Fig 1. in the main article)</th><th>δ Per atom</th><th>δ Average value</th></tr>
<tr><td>NH</td><td>7.04</td><td>7.04</td></tr>
<tr><td rowspan="2">H-8</td><td>4.15</td><td rowspan="2">3.96</td></tr>
<tr><td>3.76</td></tr>
<tr><td rowspan="2">H-15</td><td>3.10</td><td rowspan="2">3.48</td></tr>
<tr><td>3.86</td></tr>
<tr><td rowspan="2">H-11</td><td>3.42</td><td rowspan="2">3.37</td></tr>
<tr><td>3.32</td></tr>
<tr><td rowspan="2">H-18</td><td>3.40</td><td rowspan="2">3.66</td></tr>
<tr><td>3.91</td></tr>
</table>

Table S3. ^{1}H NMR chemical shifts (scaled) of the TN protons obtained from the M06-2X/6-31++G(d,p) calculations performed in DMSO for the most stable conformer TN-1 (Fig. 2 in the main article). H-6a and H-6a′ (highlighted in orange) indicate higher values, while H-6b and H-6b′ (highlighted in blue) –lower values of H-6 chemical shifts.

Atom numbers (Fig. 1 in the main article)	δ per atom
2-OH	4.22
2-OH	3.17
2'-OH	2.55

2'-OH	5.96
3-OH	4.13
3-OH	5.35
3'-OH	2.92
3'-OH	2.77
4'-OH	4.17
4'-OH	2.31
6-OH	3.99
6-OH	3.19
6'-OH	4.88
6'-OH	3.69
CH_2 crown (C-N)	3.50
CH_2 crown (C-N)	2.69
CH_2 crown (C-N)	2.76
CH_2 crown (C-N)	3.35
CH_2 crown (C-N)	4.45
CH_2 crown (C-N)	2.30
CH_2 crown (C-N)	3.56
CH_2 crown (C-N)	2.63
CH_2 crown (C-O)	3.77
CH_2 crown (C-O)	3.40
CH_2 crown (C-O)	3.53
CH_2 crown (C-O)	3.55
CH_2 crown (C-O)	3.72
CH_2 crown (C-O)	4.43
CH_2 crown (C-O)	3.51
CH_2 crown (C-O)	3.64
CH_2 crown (C-O)	3.15
CH_2 crown (C-O)	3.60
CH_2 crown (C-O)	3.70
CH_2 crown (C-O)	2.89
CH_2 crown (C-O)	3.98
CH_2 crown (C-O)	2.96
CH_2 crown (C-O)	3.64
CH_2 crown (C-O)	3.43
H-1	4.66
H-1	5.20
H-1'	4.94
H-1'	3.83
H-2	3.34
H-2	2.98
H-2'	3.05
H-2'	3.55
H-3	3.41
H-3	3.83

H-3'	3.57
H-3'	3.06
H-4	4.21
H-4	3.02
H-4'	2.69
H-4'	3.32
H-5	3.26
H-5	3.90
H-5'	3.78
H-5'	3.28
H-6a	4.03
H-6b	3.47
H-6'a	3.78
H-6'a	3.73
H-6b	3.44
H-6a	3.99
H-6'b	3.54
H-6'b	3.40

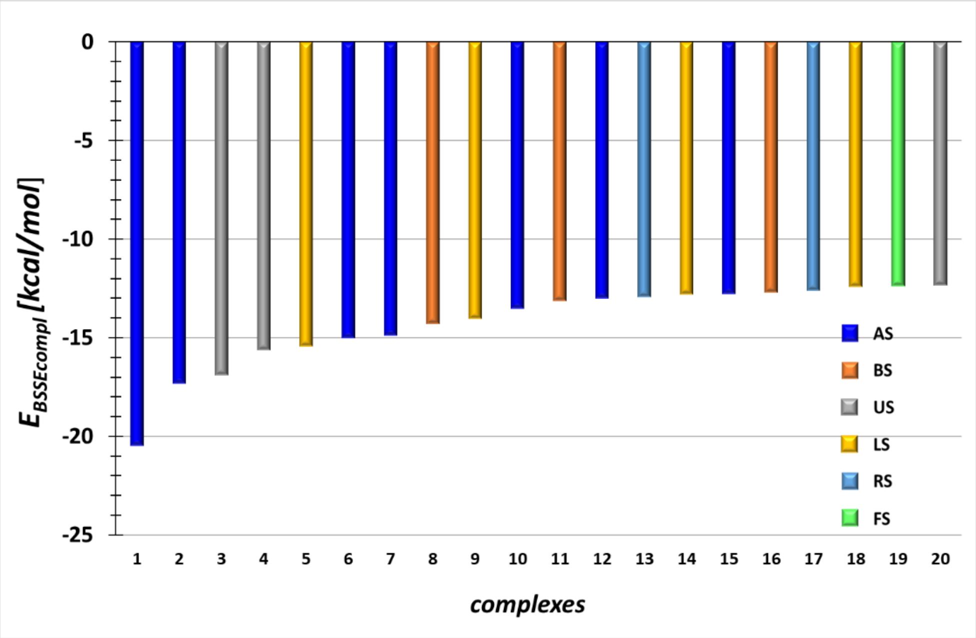

Figure S12. The BSSE corrected complexation energies ($E_{BSSEcompl}$) presented for the twenty the most energetically preferable structures of TN:BCNU, selected from different orientations (Fig. S1)

Table S4. The geometrical parameters of hydrogen bonds formed in the most stable complexes of TN:BCNU, presented in Figure 4 in the main article.

Comp.	Symb.	bond O/N-H···O	$d_{O/N-H}$ [Å]	$d_{H\cdots O}$ [Å]	$d_{O\ldots O}$ [Å]	∠ O/N-H···O [°]
**AS**	A	O29-H41···O140	0.975	1.771	2.693	156.4
	B	N142-H149···O94	1.019	2.047	3.041	164.3
**US**	A	N142-H149···O46	1.016	2.155	3.106	153.1
**RS**	A	O31-H43···O139	0.972	1.834	2.792	168.1
**FS**	A	N142-H149···O93	1.021	1.879	2.890	169.9
**BS**	A	N142-H149···O133	1.021	1.891	2.836	152.6

Table S5. The total energy (E), enthalpy (H) and corrected Gibbs energy (G_{corr}) [hartree] for the most stable complexes of TN:BCNU, presented in Figure 4 in the main article.

Complex	E	H	G_{corr}
AS	-5093.327276	-5091.916278	-5092.081060
US	-5093.323143	-5091.912644	-5092.077712
LS	-5093.316960	-5091.906609	-5092.073632
BS	-5093.318896	-5091.908210	-5092.074715
RS	-5093.313630	-5091.901853	-5092.066617
FS	-5093.315226	-5091.905297	-5092.072661

Table S6. Coordinates [Å] of the most stable complexes of TN:BCNU presented in Figure 4 in the main article.

	AS			US			LS		
Atoms	x	y	z	x	y	z	x	y	z
C	-4.3846	2.1868	2.8646	-3.4915	-5.1256	-2.2515	3.3840	-3.6476	0.6491
C	-4.4804	0.9285	3.7194	-4.1683	-3.9617	-2.9692	3.6922	-2.7960	1.8759
O	-3.1817	0.3631	3.8328	-3.1924	-2.9712	-3.2765	2.4727	-2.3026	2.4134
C	-3.0890	-1.0343	3.6223	-3.5353	-1.6362	-2.9489	2.4356	-0.9207	2.7281
C	-3.2631	-1.3732	2.1455	-3.4833	-1.4095	-1.4401	2.4542	-0.0505	1.4734
O	-2.9673	-2.7421	1.8925	-3.6859	-0.0414	-1.1223	2.1793	1.3062	1.7992
C	-4.0127	-3.6807	2.1510	-5.0381	0.4126	-1.1531	3.2936	2.0806	2.2495
C	-3.7461	-4.9200	1.3032	-5.1236	1.6557	-0.2774	3.0329	3.5396	1.8999
H	-3.5959	2.8359	3.2639	-2.5920	-5.4203	-2.8034	2.6013	-4.3738	0.8957
H	-5.3184	2.7500	2.9456	-4.1634	-5.9876	-2.2672	4.2760	-4.2250	0.3911
H	-4.8510	1.1912	4.7174	-4.6132	-4.3226	-3.9037	4.1869	-3.4218	2.6304
H	-5.1802	0.2196	3.2616	-4.9645	-3.5446	-2.3415	4.3649	-1.9733	1.6077
H	-3.8228	-1.5691	4.2407	-4.5263	-1.3835	-3.3499	3.2661	-0.6513	3.3913
H	-2.0856	-1.3254	3.9456	-2.7942	-0.9982	-3.4402	1.4911	-0.7612	3.2562
H	-2.5372	-0.8035	1.5601	-2.4833	-1.6451	-1.0717	1.6492	-0.3559	0.7994

H	-4.2707	-1.1297	1.7919	-4.2090	-2.0418	-0.9130	3.4054	-0.1260	0.9318
H	-4.9807	-3.2384	1.8777	-5.7034	-0.3705	-0.7626	4.2037	1.7316	1.7443
H	-4.0223	-3.9470	3.2140	-5.3325	0.6547	-2.1813	3.4192	1.9645	3.3320
H	-4.2879	-5.7638	1.7378	-6.0002	2.2400	-0.5673	3.6972	4.1691	2.4987
H	-2.6807	-5.1664	1.3601	-4.2467	2.2840	-0.4733	2.0080	3.8016	2.1795
C	5.2354	1.5804	-2.2753	5.8800	-1.7285	2.2859	-6.7466	-1.0155	-2.7311
C	5.6974	0.1489	-2.0433	5.8693	-0.2220	2.0770	-7.0944	0.2105	-1.9003
C	4.7115	-0.8474	-2.6298	4.6538	0.4152	2.7205	-6.1005	1.3319	-2.1278
C	3.3382	-0.5659	-2.0363	3.4123	-0.2488	2.1456	-4.7115	0.8019	-1.8101
O	2.9661	0.7864	-2.3029	3.4636	-1.6617	2.3530	-4.4240	-0.3467	-2.6105
C	3.8390	1.7243	-1.6703	4.5774	-2.2951	1.7138	-5.3179	-1.4412	-2.3806
O	5.0708	-2.1665	-2.2764	4.5870	1.7889	2.4028	-6.3551	2.4089	-1.2506
C	3.2690	3.1142	-1.8681	4.4794	-3.8050	1.8955	-4.8662	-2.6489	-3.1920
O	1.9497	3.2082	-1.3697	3.8482	-4.4684	0.8133	-4.0037	-3.5237	-2.4843
O	6.1665	2.5024	-1.7513	7.0212	-2.2970	1.6791	-7.6833	-2.0453	-2.4938
O	6.9655	-0.1055	-2.6075	7.0213	0.4023	2.5981	-8.3850	0.7035	-2.1828
O	2.3949	-1.3957	-2.6215	2.2922	0.2289	2.8077	-3.7717	1.7729	-2.1178
H	5.1751	1.7671	-3.3558	5.9400	-1.9506	3.3595	-6.8068	-0.7638	-3.7982
H	5.7188	-0.0215	-0.9544	5.7865	-0.0379	0.9926	-7.0140	-0.0742	-0.8376
H	4.6593	-0.7239	-3.7222	4.6726	0.2531	3.8089	-6.1282	1.6552	-3.1794
H	3.3752	-0.7165	-0.9464	3.3560	-0.0414	1.0641	-4.6634	0.5217	-0.7457
H	3.8875	1.5029	-0.5914	4.5561	-2.0583	0.6381	-5.3022	-1.6992	-1.3096
H	6.0248	-2.2450	-2.4187	5.4768	2.1483	2.5278	-7.3140	2.5410	-1.2394
H	3.2937	3.3746	-2.9337	3.9758	-4.0158	2.8461	-4.4086	-2.2982	-4.1245
H	3.8955	3.8295	-1.3290	5.4956	-4.2029	1.9488	-5.7544	-3.2335	-3.4438
H	1.3303	2.9343	-2.0713	2.8763	-4.4247	0.8738	-3.0838	-3.2016	-2.4663
H	6.0419	2.5777	-0.7818	6.8570	-2.3840	0.7172	-7.4506	-2.4990	-1.6573
H	7.5339	0.6368	-2.3557	7.7795	-0.1164	2.2927	-8.9800	-0.0604	-2.1796
C	1.3253	-1.8188	-1.7793	1.1000	0.3126	2.0363	-2.6045	1.8042	-1.3036
C	1.1726	-3.3294	-1.9407	0.5152	1.7105	2.2483	-2.3863	3.2498	-0.8564
O	0.0012	-3.7982	-1.2953	-0.7866	1.8074	1.6789	-1.1163	3.4167	-0.2515
C	-1.2407	-3.1650	-1.5848	-1.7436	0.8258	2.0841	0.0311	2.9767	-0.9676
C	-1.1356	-1.6542	-1.3435	-1.2101	-0.5646	1.7280	-0.1235	1.4875	-1.3010
C	0.0452	-1.0703	-2.1119	0.1325	-0.7966	2.4115	-1.4162	1.2507	-2.0727
O	0.1411	0.2929	-1.7518	0.6252	-2.0596	1.9939	-1.5651	-0.1466	-2.2848
O	-2.3472	-1.0504	-1.7270	-2.1556	-1.5312	2.0938	0.9996	1.0540	-2.0205
C	2.2969	-4.0436	-1.1996	1.3436	2.7306	1.4805	-3.3639	3.6086	0.2555
O	2.1835	-3.7712	0.1911	1.3540	2.3802	0.1064	-3.1029	2.7962	1.3908
H	1.6007	-1.6274	-0.7374	1.3566	0.2162	0.9756	-2.7771	1.1962	-0.4081
H	1.1761	-3.5894	-3.0096	0.4884	1.9472	3.3220	-2.5116	3.9195	-1.7198
H	-1.5546	-3.3682	-2.6161	-1.9502	0.9021	3.1591	0.1844	3.5708	-1.8770
H	-0.9577	-1.5198	-0.2639	-1.0646	-0.5471	0.6351	-0.1851	0.9778	-0.3253
H	-0.1604	-1.1750	-3.1901	-0.0294	-0.7886	3.5007	-1.3371	1.7753	-3.0376
H	0.9483	0.6649	-2.1469	1.5401	-2.1448	2.3174	-2.4497	-0.2945	-2.6661
H	-2.4241	-0.1942	-1.2648	-1.8841	-2.3905	1.7173	1.0155	0.0781	-2.0160
H	2.2332	-5.1214	-1.3896	0.9178	3.7307	1.6318	-3.2596	4.6710	0.5047
H	3.2665	-3.6670	-1.5381	2.3781	2.7090	1.8340	-4.3886	3.4095	-0.0730

H	1.2429	-3.8273	0.4447	0.4303	2.2806	-0.1817	-2.1402	2.7586	1.5434
C	7.0858	-1.0779	1.4740	6.5540	1.5236	-1.4993	-8.0066	-0.0134	2.0072
C	6.2826	-2.2691	1.9721	5.4340	2.4320	-1.9786	-7.1138	0.8682	2.8654
C	4.8520	-2.2247	1.4488	4.0867	1.9857	-1.4244	-5.7435	1.0455	2.2261
C	4.2384	-0.8882	1.8545	3.8705	0.5219	-1.8003	-5.1488	-0.3369	1.9794
O	5.0276	0.1491	1.3063	4.9420	-0.2378	-1.2664	-6.0255	-1.0497	1.1254
C	6.3474	0.2112	1.8246	6.2015	0.0796	-1.8412	-7.2885	-1.3277	1.7138
O	4.1666	-3.3195	2.0012	3.1110	2.8315	-1.9764	-4.9638	1.8179	3.1022
C	6.9693	1.4459	1.1779	7.1871	-0.9273	-1.2566	-8.0302	-2.1919	0.6983
O	6.0126	2.4893	1.0358	6.5837	-2.2066	-1.1051	-7.1438	-3.0888	0.0403
O	8.3626	-1.0266	2.0661	7.7798	1.8376	-2.1178	-9.2207	-0.3118	2.6565
O	6.9454	-3.4393	1.5495	5.7421	3.7474	-1.5753	-7.7682	2.1062	3.0319
O	2.9761	-0.7846	1.3063	2.7021	0.0812	-1.2175	-3.9410	-0.1876	1.3316
H	7.1605	-1.1526	0.3748	6.6263	1.6156	-0.4012	-8.1856	0.5103	1.0515
H	6.2464	-2.2237	3.0743	5.3889	2.3680	-3.0796	-6.9760	0.3715	3.8415
H	4.8667	-2.2657	0.3466	4.1048	2.0426	-0.3236	-5.8617	1.5333	1.2436
H	4.2028	-0.7928	2.9538	3.8398	0.3993	-2.8973	-5.0247	-0.8864	2.9291
H	6.3090	0.3235	2.9195	6.1441	-0.0326	-2.9355	-7.1415	-1.8804	2.6551
H	3.4465	-3.5598	1.3826	2.3636	2.8730	-1.3498	-4.2625	2.2422	2.5658
H	7.3550	1.1760	0.1847	7.5313	-0.5719	-0.2753	-8.5061	-1.5427	-0.0498
H	7.8099	1.8007	1.7790	8.0615	-1.0109	-1.9064	-8.8192	-2.7590	1.1981
H	5.1287	2.1305	1.2467	5.6254	-2.1134	-1.2696	-6.2292	-2.8462	0.2822
H	8.7647	-1.8970	1.9394	7.9117	2.7895	-2.0087	-9.5853	0.5287	2.9669
H	6.3597	-4.1766	1.7736	4.9639	4.2821	-1.7887	-7.1355	2.6886	3.4765
C	2.0093	0.0397	1.9486	2.0107	-1.0242	-1.7857	-2.9682	-1.2237	1.4250
C	0.7070	-0.7379	1.7396	0.5375	-0.6390	-1.6415	-1.6487	-0.4648	1.5670
O	-0.4355	0.0042	2.1184	-0.3077	-1.7105	-2.0325	-0.5408	-1.3505	1.5210
C	-0.5941	1.1977	1.3507	-0.2080	-2.7649	-1.1008	-0.4053	-1.8961	0.2259
C	0.5957	2.1097	1.6563	1.1964	-3.3589	-1.0747	-1.6214	-2.7340	-0.1657
C	1.8943	1.3961	1.2641	2.3018	-2.2925	-0.9724	-2.9676	-2.0497	0.1338
O	3.0131	2.2085	1.5734	3.5463	-2.8277	-1.3826	-4.0070	-3.0084	0.1999
O	0.5023	3.3519	0.9852	1.2498	-4.2625	0.0278	-1.4918	-3.0289	-1.5560
C	0.6930	-2.0761	2.4575	0.0981	0.6241	-2.3589	-1.5292	0.4227	2.7935
O	-0.1284	-3.0199	1.7908	-0.9715	1.2513	-1.6615	-0.7255	1.5614	2.5214
H	2.2508	0.1645	3.0135	2.2940	-1.1701	-2.8369	-3.1640	-1.8681	2.2924
H	0.6572	-0.9317	0.6636	0.3925	-0.4400	-0.5725	-1.6035	0.2086	0.7024
H	-0.6244	0.9781	0.2758	-0.4195	-2.3889	-0.0928	-0.3167	-1.0867	-0.5077
H	0.6043	2.3207	2.7341	1.3592	-3.9389	-1.9901	-1.5921	-3.6858	0.3752
H	1.8477	1.1998	0.1782	2.3363	-1.9742	0.0826	-3.1441	-1.3318	-0.6837
H	2.7720	3.1171	1.3380	3.7820	-3.5275	-0.7389	-4.0895	-3.4006	-0.6941
H	0.9278	3.2818	0.1054	0.6054	-3.9626	0.6936	-1.0764	-2.2563	-1.9794
H	1.7086	-2.4802	2.4581	0.9237	1.3421	-2.3660	-2.5210	0.7984	3.0560
H	0.3776	-1.9232	3.4974	-0.1759	0.3850	-3.3948	-1.1378	-0.1633	3.6346
H	-1.0546	-2.9183	2.0651	-1.8098	0.7840	-1.8113	0.2111	1.3120	2.4568
N	-4.1605	1.9160	1.4452	-3.1598	-4.8651	-0.8515	3.0071	-2.8825	-0.5386
N	-4.1473	-4.8117	-0.0964	-5.2257	1.3874	1.1554	3.2515	3.8679	0.4937
C	-5.3153	2.0756	0.5613	-4.0245	-5.4738	0.1543	4.0115	-2.7646	-1.5904

C	-6.4208	1.0536	0.8206	-5.4268	-4.8667	0.1676	5.1687	-1.8462	-1.2080
O	-6.0020	-0.2865	0.6868	-5.4296	-3.4705	0.3657	4.7517	-0.5369	-0.8734
C	-5.6081	-0.5933	-0.6415	-5.0401	-3.0952	1.6773	4.2284	0.1930	-1.9751
C	-5.2018	-2.0382	-0.7453	-5.1299	-1.5968	1.8132	3.9132	1.5822	-1.4896
O	-6.2888	-2.8901	-0.4231	-6.4771	-1.1697	1.6893	5.1220	2.2079	-1.0867
C	-6.3098	-4.1098	-1.1406	-6.8067	-0.0391	2.4739	5.1281	3.6225	-1.1631
C	-5.5012	-5.2172	-0.4640	-6.5558	1.2860	1.7529	4.5852	4.3050	0.0897
C	-2.9383	1.6438	0.9009	-2.0453	-4.1934	-0.4514	1.7551	-2.3869	-0.7548
C	-3.3062	-4.4141	-1.1082	-4.1438	1.2999	1.9924	2.2419	3.9044	-0.4381
H	-4.9569	2.0266	-0.4667	-3.5361	-5.3612	1.1207	3.5098	-2.4090	-2.4891
H	-5.7340	3.0786	0.7126	-4.1140	-6.5464	-0.0546	4.4182	-3.7595	-1.8053
H	-7.2489	1.2696	0.1285	-6.0170	-5.3717	0.9463	5.8897	-1.8189	-2.0359
H	-6.8078	1.1538	1.8398	-5.9260	-5.0411	-0.7910	5.6921	-2.2312	-0.3258
H	-4.7406	0.0069	-0.9358	-4.0007	-3.3895	1.8746	3.3066	-0.2691	-2.3469
H	-6.4392	-0.3753	-1.3311	-5.6948	-3.5858	2.4127	4.9752	0.2256	-2.7828
H	-4.3455	-2.2216	-0.0878	-4.4927	-1.1290	1.0548	3.2060	1.5161	-0.6548
H	-4.8514	-2.2179	-1.7707	-4.7175	-1.3115	2.7894	3.4252	2.1517	-2.2894
H	-5.9449	-3.9532	-2.1614	-6.2531	-0.0605	3.4186	4.5751	3.9517	-2.0472
H	-7.3536	-4.4358	-1.1933	-7.8760	-0.1017	2.6997	6.1741	3.9274	-1.2742
H	-5.4804	-6.0916	-1.1254	-6.7291	2.1095	2.4561	4.6163	5.3914	-0.0677
H	-6.0044	-5.5057	0.4614	-7.2721	1.3839	0.9348	5.2533	4.0706	0.9223
N	-1.8470	1.7768	1.7430	-1.2035	-3.7478	-1.4422	0.8082	-2.6689	0.2026
N	-2.1864	-3.7417	-0.6899	-2.9345	1.1160	1.3611	1.1317	3.1721	-0.0852
O	-3.5667	-4.6202	-2.2932	-4.2517	1.3796	3.2163	2.3381	4.5193	-1.4980
O	-2.7954	1.3669	-0.2995	-1.7638	-4.0155	0.7488	1.4560	-1.7487	-1.7788
H	-2.0343	1.6287	2.7313	-1.5967	-3.6128	-2.3705	1.1440	-2.8937	1.1354
H	-2.2077	-3.3392	0.2406	-2.9623	0.7580	0.4090	1.2965	2.4181	0.5769
Cl	-4.5258	1.2750	-3.2727	-3.4772	2.3219	-3.4620	7.6804	-3.4418	1.2830
Cl	-0.6478	6.5565	1.7157	-1.2849	5.2693	3.0866	7.8735	-0.5639	-3.4962
O	-4.0191	4.4683	-1.3439	-3.7363	5.1660	-1.1066	9.4031	0.0053	0.5693
O	-0.2233	2.8282	-2.9139	0.3354	3.8318	-2.1818	5.5974	-0.1928	2.7807
N	-2.0977	3.5542	-2.1951	-1.7074	4.3251	-1.7598	7.4022	-0.3584	1.6388
N	-2.1755	4.3949	-0.0078	-2.4658	4.0980	0.4477	7.4811	0.6206	-0.4877
N	-0.8352	3.3007	-1.9618	-0.5098	3.9596	-1.3188	6.1788	0.1446	1.7656
C	-2.6713	3.2911	-3.5091	-1.9486	4.5775	-3.1745	8.0231	-1.1258	2.7124
C	-2.8462	5.0314	1.1099	-3.3803	4.4153	1.5298	8.1642	1.3503	-1.5384
C	-2.8581	4.1665	-1.1368	-2.7276	4.5629	-0.7839	8.1885	0.1031	0.5225
C	-2.8718	1.7983	-3.7513	-2.0418	3.2866	-3.9679	7.4700	-2.5352	2.8247
C	-1.8554	5.3146	2.2178	-2.7280	4.2053	2.8798	8.9556	0.4706	-2.4900
H	-1.2627	3.9717	0.1551	-1.7133	3.4355	0.6140	6.4634	0.6298	-0.4550
H	-3.6065	3.8436	-3.5653	-2.8659	5.1589	-3.2407	9.0919	-1.1321	2.5076
H	-1.9669	3.6834	-4.2456	-1.1103	5.1634	-3.5598	7.8314	-0.6064	3.6559
H	-3.3266	5.9531	0.7715	-3.7028	5.4527	1.4161	7.4019	1.9153	-2.0776
H	-3.6316	4.3793	1.5125	-4.2799	3.7858	1.4916	8.8737	2.0556	-1.0904
H	-2.1704	1.1918	-3.1752	-1.1713	2.6557	-3.7997	6.4057	-2.5311	3.0493
H	-2.7739	1.5699	-4.8111	-2.1596	3.5021	-5.0285	8.0080	-3.0830	3.5970
H	-2.3660	5.7047	3.0964	-3.4268	4.4520	3.6770	9.5311	1.0838	-3.1825

H	-1.2880	4.4190	2.4755	-2.3760	3.1838	3.0080	9.6186	-0.1989	-1.9448
	**BS**			**RS**			**FS**		
**Atoms**	**x**	**y**	**z**	**x**	**y**	**z**	**x**	**y**	**z**
C	3.5066	-5.3492	-1.4090	6.9111	-3.3410	1.4103	-4.3093	3.8636	-0.1820
C	3.9627	-5.3820	0.0462	7.2131	-2.1907	2.3644	-4.8400	3.3229	1.1418
O	2.8505	-5.0934	0.8884	5.9916	-1.7201	2.9262	-3.7656	2.7310	1.8649
C	3.1050	-4.1919	1.9520	5.8402	-0.3117	2.9691	-4.0334	1.4664	2.4447
C	3.1448	-2.7589	1.4281	5.4987	0.2320	1.5840	-4.0524	0.3747	1.3774
O	3.2691	-1.8147	2.4803	5.2319	1.6248	1.6253	-4.1544	-0.9183	1.9553
C	4.5653	-1.7117	3.0743	6.3735	2.4725	1.7623	-5.4540	-1.2879	2.4206
C	4.7010	-0.3222	3.6897	6.0142	3.8424	1.1979	-5.5466	-2.8094	2.4118
H	2.5713	-5.9102	-1.5114	6.2136	-4.0370	1.8894	-3.3929	4.4342	0.0042
H	4.2489	-5.8646	-2.0239	7.8328	-3.8988	1.2258	-5.0369	4.5668	-0.5954
H	4.3415	-6.3823	0.2870	7.8612	-2.5486	3.1729	-5.2530	4.1497	1.7317
H	4.7668	-4.6530	0.2009	7.7303	-1.3866	1.8279	-5.6359	2.5922	0.9563
H	4.0406	-4.4546	2.4630	6.7491	0.1596	3.3660	-4.9798	1.4906	3.0014
H	2.2826	-4.3056	2.6641	5.0182	-0.1071	3.6612	-3.2227	1.2729	3.1531
H	2.1909	-2.5330	0.9465	4.5721	-0.2293	1.2361	-3.0995	0.3790	0.8462
H	3.9507	-2.6357	0.6918	6.2982	0.0074	0.8649	-4.8602	0.5420	0.6531
H	5.3409	-1.8619	2.3100	7.2222	2.0477	1.2074	-6.2202	-0.8626	1.7569
H	4.6764	-2.4720	3.8558	6.6439	2.5660	2.8203	-5.6062	-0.9103	3.4381
H	5.4805	-0.3553	4.4539	6.6910	4.5858	1.6257	-6.3369	-3.1157	3.1012
H	3.7674	-0.0693	4.2031	5.0046	4.1063	1.5295	-4.6107	-3.2233	2.8013
C	-5.2327	1.1639	-2.5832	-3.3327	-2.4085	-2.5078	4.7615	-1.4622	-3.1398
C	-5.4761	1.7101	-1.1845	-3.9439	-1.0809	-2.0795	4.9214	-2.5715	-2.1110
C	-4.2608	2.4470	-0.6573	-3.0060	0.1083	-2.2893	3.7031	-3.4728	-2.0713
C	-3.0765	1.4942	-0.7030	-1.5941	-0.1828	-1.8043	2.4824	-2.6071	-1.8026
O	-2.8831	1.0110	-2.0330	-1.1239	-1.3680	-2.4397	2.3728	-1.5819	-2.7923
C	-3.9962	0.2624	-2.5306	-1.8724	-2.5231	-2.0501	3.4866	-0.6835	-2.8032
O	-4.4528	2.8215	0.6907	-3.4662	1.2494	-1.5761	3.8051	-4.3991	-1.0104
C	-3.6450	-0.3175	-3.8933	-1.2265	-3.7717	-2.6410	3.2213	0.4504	-3.7882
O	-3.0985	-1.6233	-3.8273	-0.3247	-4.4276	-1.7668	2.7481	1.6365	-3.1675
O	-6.3776	0.4761	-3.0426	-4.0738	-3.4916	-1.9907	5.9059	-0.6353	-3.1450
O	-6.5810	2.5850	-1.1323	-5.1445	-0.8830	-2.8003	6.0547	-3.3737	-2.3557
O	-1.9328	2.1753	-0.3248	-0.7820	0.8732	-2.1820	1.3438	-3.3908	-1.8774
H	-5.0550	1.9958	-3.2774	-3.3742	-2.4778	-3.6029	4.6743	-1.8997	-4.1429
H	-5.6357	0.8469	-0.5168	-4.1654	-1.1651	-1.0073	4.9928	-2.0933	-1.1194
H	-4.0412	3.3196	-1.2910	-2.9426	0.3267	-3.3641	3.5753	-3.9801	-3.0394
H	-3.2713	0.6398	-0.0353	-1.5679	-0.3308	-0.7096	2.5806	-2.1390	-0.8103
H	-4.2145	-0.5633	-1.8342	-1.8667	-2.5920	-0.9526	3.6177	-0.2620	-1.7938
H	-5.3428	3.1966	0.7527	-4.3700	1.4579	-1.8601	4.7033	-4.7582	-1.0371
H	-2.9713	0.3759	-4.4109	-0.7438	-3.5047	-3.5884	2.5284	0.0977	-4.5595
H	-4.5676	-0.4000	-4.4729	-2.0234	-4.4893	-2.8465	4.1686	0.7102	-4.2675
H	-2.1580	-1.6191	-3.5745	0.5612	-4.0245	-1.7608	1.7759	1.6721	-3.1155
H	-6.3967	-0.4135	-2.6327	-3.9394	-3.4885	-1.0240	5.8553	-0.0174	-2.3867
H	-7.3020	2.1477	-1.6080	-5.6652	-0.2249	-2.3109	6.7909	-2.7688	-2.5266
C	-0.9346	1.4058	0.3370	0.3687	1.1313	-1.3848	0.2557	-2.9962	-1.0481

C	-0.4821	2.2147	1.5537	0.4929	2.6546	-1.2796	-0.2446	-4.2514	-0.3312
O	0.6977	1.6824	2.1344	1.7410	3.0500	-0.7402	-1.4801	-4.0230	0.3235
C	1.8060	1.4948	1.2641	2.9246	2.5168	-1.3258	-2.5435	-3.4776	-0.4488
C	1.3866	0.5033	0.1756	2.8585	0.9882	-1.2682	-2.0880	-2.1338	-1.0282
C	0.2223	1.1019	-0.5982	1.6130	0.5035	-1.9989	-0.8318	-2.3246	-1.8707
O	-0.1458	0.1931	-1.6190	1.5964	-0.9079	-1.9079	-0.4164	-1.0479	-2.3273
O	2.4698	0.2278	-0.6675	4.0292	0.4510	-1.8294	-3.1284	-1.5628	-1.7731
C	-1.5178	2.1221	2.6634	-0.5167	3.2063	-0.2791	0.7047	-4.6182	0.8018
O	-1.6219	0.7767	3.1067	-0.1847	2.7546	1.0259	0.6963	-3.5841	1.7754
H	-1.3835	0.4702	0.6890	0.2082	0.7265	-0.3762	0.6234	-2.2949	-0.2903
H	-0.3278	3.2598	1.2485	0.3318	3.0906	-2.2766	-0.3240	-5.0735	-1.0577
H	2.1236	2.4492	0.8277	3.0436	2.8655	-2.3588	-2.8524	-4.1671	-1.2440
H	1.0617	-0.4035	0.7138	2.7756	0.7305	-0.1990	-1.8437	-1.5097	-0.1511
H	0.5740	2.0486	-1.0403	1.6814	0.8216	-3.0518	-1.0872	-2.9733	-2.7236
H	-0.9341	0.5535	-2.0646	0.7753	-1.2283	-2.3218	0.4421	-1.1536	-2.7744
H	2.2150	-0.4781	-1.2924	4.0020	-0.5196	-1.7450	-2.8733	-0.6497	-2.0065
H	-1.2276	2.7845	3.4879	-0.5137	4.3012	-0.3227	0.3957	-5.5727	1.2432
H	-2.4972	2.4239	2.2797	-1.5199	2.8396	-0.5206	1.7252	-4.7073	0.4163
H	-0.7290	0.4026	3.2203	0.7846	2.7861	1.1247	-0.2244	-3.3204	1.9571
C	-7.0557	-0.4230	1.8143	-4.0763	-0.5133	1.4353	6.1409	-1.9266	1.6888
C	-6.1689	-0.4735	3.0484	-3.7363	0.8566	1.9995	5.1483	-2.4164	2.7310
C	-4.7004	-0.3511	2.6648	-2.2286	0.8662	2.2081	3.7276	-2.3823	2.1843
C	-4.3742	-1.4498	1.6580	-1.6912	-0.5116	2.6851	3.4311	-0.9648	1.7057
O	-5.2155	-1.2843	0.5306	-2.6395	-1.4109	3.1853	4.3765	-0.6244	0.7067
C	-6.5933	-1.4809	0.8161	-3.8918	-1.5702	2.5085	5.7109	-0.5501	1.1902
O	-3.9421	-0.4600	3.8422	-1.8184	1.8929	3.0797	2.8664	-2.7904	3.2157
C	-7.3085	-1.3772	-0.5291	-4.0319	-2.9775	1.9462	6.5480	-0.0611	0.0114
O	-6.5210	-1.9329	-1.5751	-3.2446	-3.1932	0.7920	5.8334	0.8935	-0.7645
O	-8.4040	-0.6782	2.1306	-5.3899	-0.6037	0.9476	7.4407	-1.8151	2.2188
O	-6.5652	0.5747	3.9037	-4.1455	1.8873	1.1190	5.5252	-3.7236	3.0998
O	-3.0683	-1.2918	1.2453	-0.9920	-1.0699	1.5844	2.1760	-0.9446	1.1331
H	-6.9370	0.5720	1.3501	-3.3516	-0.7261	0.6316	6.1164	-2.6296	0.8376
H	-6.3165	-1.4501	3.5410	-4.2598	1.0269	2.9474	5.1987	-1.7398	3.6015
H	-4.5387	0.6163	2.1601	-1.7763	1.0262	1.2202	3.6624	-3.0469	1.3062
H	-4.5342	-2.4495	2.0986	-1.0078	-0.3466	3.5236	3.4972	-0.2453	2.5409
H	-6.7386	-2.4815	1.2528	-4.6783	-1.4271	3.2619	5.7594	0.1714	2.0213
H	-3.0849	-0.0159	3.6786	-1.2660	2.4862	2.5428	2.0479	-3.1245	2.7946
H	-7.5101	-0.3203	-0.7531	-5.0752	-3.1233	1.6531	6.8137	-0.9161	-0.6256
H	-8.2687	-1.8963	-0.4798	-3.7880	-3.7016	2.7332	7.4764	0.3853	0.3756
H	-5.6336	-2.1296	-1.2182	-2.2967	-3.2086	1.0333	4.9121	0.9142	-0.4443
H	-8.6385	-0.0822	2.8553	-5.6061	0.2589	0.5535	7.6421	-2.6596	2.6450
H	-5.9161	0.5986	4.6212	-3.7380	1.7078	0.2508	4.8193	-4.0583	3.6711
C	-2.3600	-2.4112	0.7245	0.2492	-1.7037	1.8508	1.4646	0.2866	1.0838
C	-0.9607	-2.2545	1.3232	1.3966	-0.6863	1.7587	0.0231	-0.1069	1.4154
O	-0.0607	-3.2073	0.7781	2.6365	-1.3653	1.9358	-0.8640	0.9896	1.2426
C	0.1729	-2.9164	-0.5804	2.9103	-2.1519	0.7989	-0.9550	1.3164	-0.1263
C	-1.0973	-3.0771	-1.4080	1.8658	-3.2490	0.6143	0.3786	1.8260	-0.6574

C	-2.2924	-2.3190	-0.8054	0.4381	-2.7013	0.7084	1.5433	0.8798	-0.3265
O	-3.5081	-2.7943	-1.3532	-0.5048	-3.7427	0.8634	2.7884	1.5485	-0.4770
O	-0.8204	-2.6133	-2.7268	2.0808	-3.8525	-0.6583	0.2396	2.0008	-2.0631
C	-0.8732	-2.2976	2.8376	1.3980	0.5302	2.6664	-0.1947	-0.7106	2.7907
O	0.1862	-1.4720	3.3054	2.2219	1.5504	2.1168	-1.2598	-1.6511	2.7667
H	-2.8334	-3.3535	1.0314	0.2340	-2.2194	2.8206	1.8643	0.9978	1.8165
H	-0.6310	-1.2497	1.0335	1.3581	-0.3005	0.7332	-0.2410	-0.8934	0.6980
H	0.5011	-1.8752	-0.6885	2.8862	-1.5257	-0.1008	-1.2167	0.4241	-0.7073
H	-1.3542	-4.1421	-1.4627	2.0006	-4.0059	1.3979	0.5808	2.8074	-0.2044
H	-2.1427	-1.2529	-1.0423	0.2449	-2.1377	-0.2195	1.4786	0.0339	-1.0284
H	-3.5010	-2.5552	-2.3027	-0.5577	-4.2046	-0.0009	2.9035	1.7360	-1.4396
H	0.0009	-2.0885	-2.7030	2.7993	-3.3777	-1.1147	-0.4816	1.4209	-2.3692
H	-1.7930	-1.8854	3.2589	0.3977	0.9614	2.7207	0.7014	-1.2649	3.0800
H	-0.7578	-3.3347	3.1740	1.7159	0.2494	3.6776	-0.3718	0.0874	3.5225
H	1.0440	-1.8720	3.0985	3.1554	1.3926	2.3207	-2.1120	-1.1975	2.6722
N	3.3489	-4.0061	-1.9646	6.3913	-2.9320	0.1063	-4.0714	2.8443	-1.2029
N	5.0513	0.7464	2.7562	6.0982	3.9567	-0.2550	-5.8420	-3.4040	1.1113
C	4.4176	-3.5263	-2.8343	7.3269	-2.9588	-1.0126	-5.0647	2.7382	-2.2662
C	5.7338	-3.3332	-2.0812	8.4265	-1.9072	-0.8685	-6.4158	2.2433	-1.7515
O	5.6177	-2.4811	-0.9651	7.9261	-0.6007	-0.6954	-6.3347	1.0084	-1.0758
C	5.3679	-1.1310	-1.3294	7.2919	-0.0926	-1.8587	-6.0578	-0.0791	-1.9435
C	5.3414	-0.2762	-0.0908	6.8949	1.3413	-1.6197	-6.0491	-1.3601	-1.1500
O	6.6115	-0.2819	0.5469	8.0488	2.1352	-1.3958	-7.3365	-1.6152	-0.6111
C	6.9620	0.9522	1.1412	7.9486	3.4637	-1.8722	-7.6793	-2.9865	-0.5428
C	6.4625	1.0876	2.5812	7.3482	4.4266	-0.8469	-7.2379	-3.6537	0.7611
C	2.2353	-3.2467	-1.7906	5.0820	-2.6504	-0.1263	-2.9494	2.0776	-1.2610
C	4.1255	1.4868	2.0730	5.0434	3.7195	-1.1000	-4.8779	-3.8167	0.2256
H	4.0807	-2.5963	-3.2890	6.7534	-2.8093	-1.9259	-4.6631	2.0724	-3.0281
H	4.5804	-4.2551	-3.6373	7.7953	-3.9487	-1.0667	-5.2074	3.7250	-2.7226
H	6.4845	-2.9501	-2.7881	9.0785	-1.9612	-1.7525	-7.1103	2.1778	-2.6018
H	6.0987	-4.2926	-1.6998	9.0435	-2.1179	0.0111	-6.8360	2.9576	-1.0361
H	4.3955	-1.0303	-1.8268	6.3818	-0.6629	-2.0890	-5.0703	0.0351	-2.4101
H	6.1505	-0.7809	-2.0191	7.9812	-0.1605	-2.7136	-6.8216	-0.1249	-2.7340
H	4.5622	-0.6431	0.5848	6.2072	1.3900	-0.7688	-5.2947	-1.2896	-0.3591
H	5.0527	0.7390	-0.3824	6.3364	1.6980	-2.4946	-5.7324	-2.1759	-1.8119
H	6.5860	1.7833	0.5347	7.3634	3.4905	-2.7972	-7.2571	-3.5238	-1.3984
H	8.0554	1.0062	1.1598	8.9649	3.8036	-2.0967	-8.7708	-3.0502	-0.6000
H	6.6587	2.1090	2.9278	7.2110	5.4047	-1.3237	-7.4360	-4.7299	0.6877
H	7.0276	0.4016	3.2152	8.0559	4.5488	-0.0243	-7.8403	-3.2556	1.5803
N	1.2285	-3.7893	-1.0320	4.2344	-2.7023	0.9502	-1.9998	2.2916	-0.2923
N	2.8589	0.9667	2.0646	4.0083	3.0058	-0.5446	-3.6280	-3.2940	0.4544
O	4.4392	2.5357	1.4969	5.0482	4.0933	-2.2726	-5.1311	-4.5693	-0.7145
O	2.0928	-2.1247	-2.3200	4.6370	-2.3979	-1.2656	-2.7497	1.2453	-2.1673
H	1.4739	-4.5126	-0.3597	4.6276	-2.6032	1.8836	-2.2775	2.7516	0.5707
H	2.7726	-0.0239	2.2772	4.2107	2.4736	0.2973	-3.5758	-2.4582	1.0298
Cl	-0.1536	2.8806	-3.4627	-7.2410	1.6628	2.3287	-0.8772	5.2355	0.8382
Cl	1.7094	4.7704	2.3915	-8.7734	-2.0612	0.2245	1.1081	3.0714	3.2749

O	2.2958	5.3526	-2.1206	-6.3248	1.0703	-1.1928	2.3675	6.2734	1.1715
O	4.2138	1.4126	-2.6483	-9.3822	4.0136	0.0549	1.7068	4.3676	-2.7566
N	3.0812	3.2144	-2.3705	-7.8524	2.6063	-0.4676	1.8207	5.2367	-0.7977
N	3.3923	4.3985	-0.3683	-8.5288	0.6010	-1.4666	2.9243	4.0792	0.9116
N	3.9788	2.3362	-1.9023	-9.1449	2.9693	-0.5016	2.2506	4.3208	-1.6758
C	2.5786	3.0924	-3.7342	-6.8472	3.3959	0.2371	0.7605	6.1764	-1.1356
C	3.3997	5.6256	0.4039	-8.2918	-0.6894	-2.0999	3.5045	3.9597	2.2351
C	2.8847	4.4132	-1.6115	-7.5189	1.3738	-1.0714	2.4035	5.2483	0.5119
C	1.4295	2.1019	-3.8453	-7.0214	3.3518	1.7507	-0.6037	5.5385	-0.9217
C	2.0713	5.9407	1.0687	-7.6780	-1.7222	-1.1669	2.5128	4.1989	3.3655
H	3.8239	3.5663	0.0358	-9.4758	0.9129	-1.3012	2.8677	3.2468	0.3231
H	2.2851	4.0878	-4.0616	-5.8785	2.9863	-0.0353	0.8944	7.0567	-0.5092
H	3.4082	2.7342	-4.3479	-6.9264	4.4326	-0.0988	0.8707	6.4474	-2.1867
H	4.1871	5.5405	1.1548	-9.2525	-1.0418	-2.4745	3.9341	2.9581	2.3095
H	3.6398	6.4617	-0.2603	-7.6241	-0.5665	-2.9583	4.3133	4.6884	2.3601
H	1.5423	1.2677	-3.1494	-7.8929	3.9120	2.0807	-0.6782	4.5681	-1.4196
H	1.3458	1.7277	-4.8643	-6.1190	3.7377	2.2226	-1.4007	6.1987	-1.2615
H	2.0925	6.9309	1.5223	-7.5286	-2.6604	-1.7006	2.9933	4.0338	4.3289
H	1.2524	5.8776	0.3522	-6.7333	-1.3930	-0.7355	2.1020	5.2047	3.3160

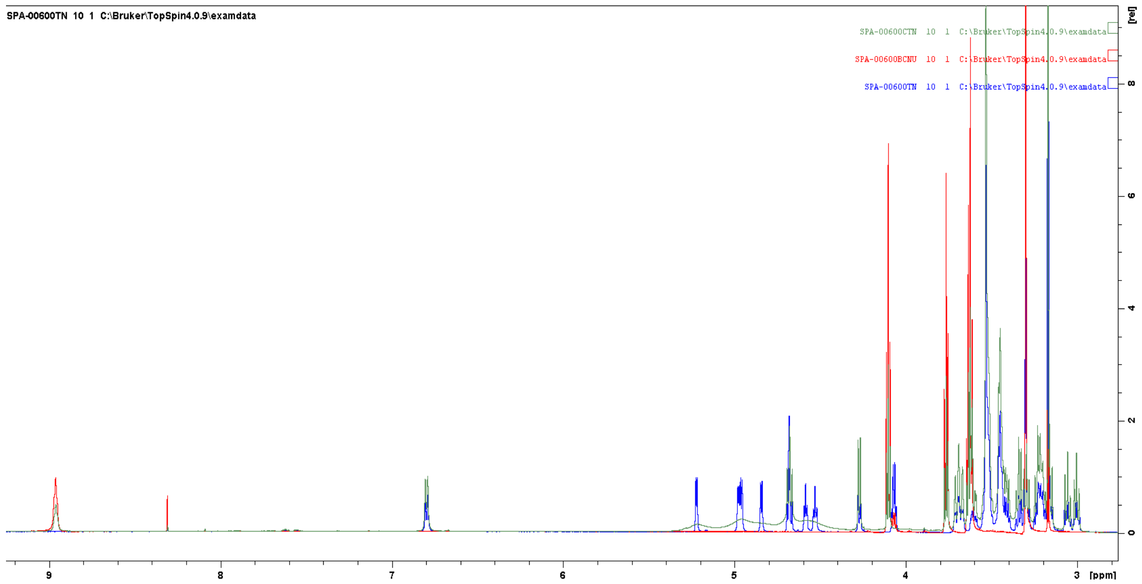

Figure S13. Overlapping the ^{1}H NMR spectra of the complex (green); BCNU (red) and TN (blue) measured in DMSO-d$_6$.

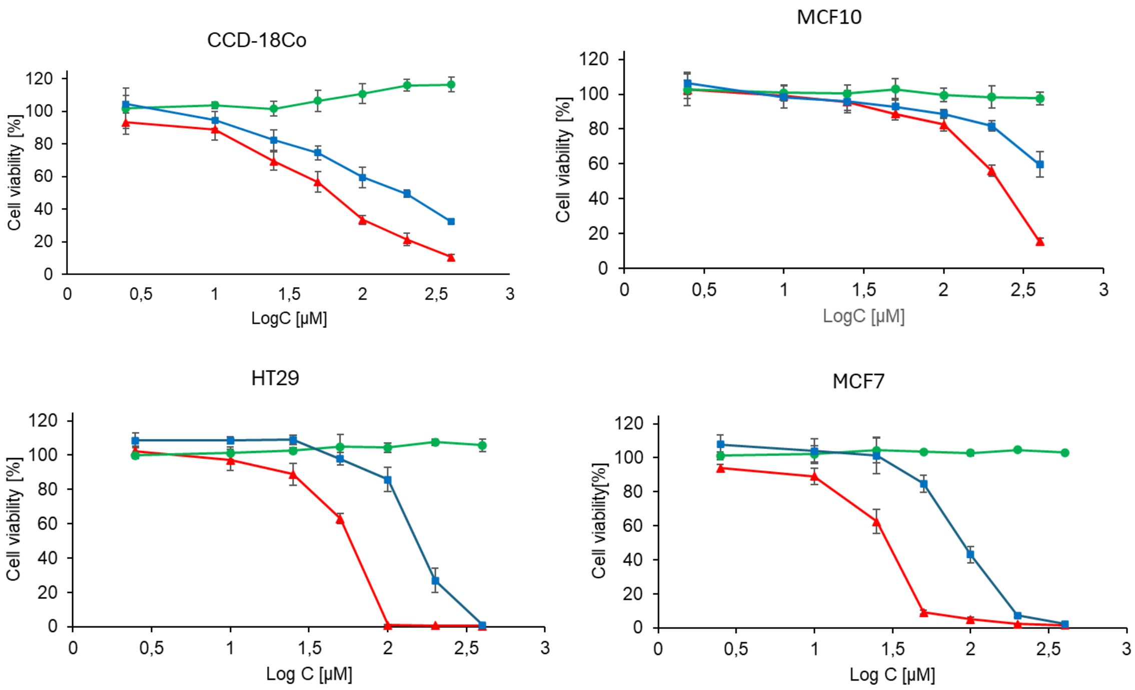

Figure S14. Effect of BCNU, TN and TN:BCNU complex on viability of normal and cancer cells. Human colon normal CCD-18Co and cancer HT29 cells as well as breast normal and cancer cells, i.e. MCF10A and MCF-7 were treated for 24 h with different concentrations of TN (green), BCNU (red) and TN:BCNU complex (blue line).

References:

1. HyperChem(TM) Professional 8.0, Hypercube, Inc., 1115 NW 4th Street, Gainesville, Florida 32601, USA.

2. Porwanski, S.; Dumarcay-Charbonnier, F.; Menuel, S.; Joly, J.-P.; Bulach, V.; Marsura, A. Bis-β-Cyclodextrinyl- and Bis-Cellobiosyl-Diazacrowns: Synthesis and Molecular Complexation Behaviors toward Busulfan Anticancer Agent and Two Basic Aminoacids. *Tetrahedron* **2009**, *65*, 6196–6203, doi:10.1016/j.tet.2009.05.057.

3. Knox, C.; Wilson, M.; Klinger, C.M.; Franklin, M.; Oler, E.; Wilson, A.; Pon, A.; Cox, J.; Chin, N.E. (Lucy); Strawbridge, S.A.; et al. DrugBank 6.0: The DrugBank Knowledgebase for 2024. *Nucleic Acids Res* **2024**, *52*, D1265–D1275, doi:10.1093/nar/gkad976.

4. Schlesinger, C.; Alig, E.; Schmidt, M.U. Crystal Structure of the Anticancer Drug Carmustine Determined by X-Ray Powder Diffraction. *Powder Diffr* **2021**, *36*, 148–150, doi:10.1017/S0885715621000294.

5. Adamiak, M.; Ignaczak, A. DFT Studies on the Physicochemical Properties of a New Potential Drug Carrier Containing Cellobiose Units and Its Complex with Paracetamol. *Struct Chem* **2022**, *33*, 1365–1378, doi:10.1007/s11224-022-01950-y.

6. Adamiak, M.; Ignaczak, A. Quantum Chemical Study of the Complexation Process of Bis-β-d-Glucopyranosyl Diazacrown Derivative with Aspirin and Paracetamol Molecules. *Comput Theor Chem* **2019**, *1167*, 112591, doi:10.1016/j.comptc.2019.112591.

7. Adamiak, M.; Porwański, S.; Ignaczak, A. Conformational Search and Spectroscopic Analysis of Bis -β- d -Glucopyranosyl Azacrown Derivative. *Tetrahedron* **2018**, *74*, 2166–2173, doi:10.1016/j.tet.2018.03.025.

8. Hocquet, A.; Langgård, M. An Evaluation of the MM+ Force Field. *J Mol Model* **1998**, *4*, 94–112, doi:10.1007/s008940050128.

9. Weiner, S.J.; Kollman, P.A.; Case, D.A.; Singh, U.C.; Ghio, C.; Alagona, G.; Profeta, S.; Weiner, P. A New Force Field for Molecular Mechanical Simulation of Nucleic Acids and Proteins. *J Am Chem Soc* **1984**, *106*, 765–784, doi:10.1021/ja00315a051.

10. Weiner, S.J.; Kollman, P.A.; Nguyen, D.T.; Case, D.A. An All Atom Force Field for Simulations of Proteins and Nucleic Acids. *J Comput Chem* **1986**, *7*, 230–252, doi:10.1002/jcc.540070216.

11. Brooks, B.R.; Bruccoleri, R.E.; Olafson, B.D.; States, D.J.; Swaminathan, S.; Karplus, M. CHARMM: A Program for Macromolecular Energy, Minimization, and Dynamics Calculations. *J Comput Chem* **1983**, *4*, 187–217, doi:10.1002/jcc.540040211.

12. Stewart, J.J.P. Optimization of Parameters for Semiempirical Methods VI: More Modifications to the NDDO Approximations and Re-Optimization of Parameters. *J Mol Model* **2013**, *19*, 1–32, doi:10.1007/s00894-012-1667-x.

13. James J. P. Stewart MOPAC2016 **2016**. Stewart Computational Chemistry, Colorado Springs, CO, USA, https://OpenMOPAC.net.

14. Guarnieri, F.; Still, W.C. A Rapidly Convergent Simulation Method: Mixed Monte Carlo/Stochastic Dynamics. *J Comput Chem* **1994**, *15*, 1302–1310, doi:10.1002/jcc.540151111.

15. Frisch, M.J.; T.G.W.; S.H.B.; S.G.E.; et al. Gaussian 16, Revision C.01 **2016**.

16. Zhao, Y.; Truhlar, D.G. The M06 Suite of Density Functionals for Main Group Thermochemistry, Thermochemical Kinetics, Noncovalent Interactions, Excited States, and Transition Elements: Two New Functionals and Systematic Testing of Four M06-Class Functionals and 12 Other Functionals. *Theor Chem Acc* **2008**, *120*, 215–241, doi:10.1007/s00214-007-0310-x.

17. Grimme, S.; Antony, J.; Ehrlich, S.; Krieg, H. A Consistent and Accurate *Ab Initio* Parametrization of Density Functional Dispersion Correction (DFT-D) for the 94 Elements H-Pu. *J Chem Phys* **2010**, *132*, doi:10.1063/1.3382344.

18. Rassolov, V.A.; Ratner, M.A.; Pople, J.A.; Redfern, P.C.; Curtiss, L.A. 6-31G* Basis Set for Third-row Atoms. *J Comput Chem* **2001**, *22*, 976–984, doi:10.1002/jcc.1058.

19. Mardirossian, N.; Head-Gordon, M. How Accurate Are the Minnesota Density Functionals for Noncovalent Interactions, Isomerization Energies, Thermochemistry, and Barrier Heights Involving Molecules Composed of Main-Group Elements? *J Chem Theory Comput* **2016**, *12*, 4303–4325, doi:10.1021/acs.jctc.6b00637.

20. Funes-Ardoiz, I.; Robert, S. *Paton GoodVibes*, Version 2.0.3 (v2.0.3); 2018; Zenodo https://doi.org/10.5281/zenodo.1435820

21. Luchini, G.; Alegre-Requena, J. V.; Funes-Ardoiz, I.; Paton, R.S. GoodVibes: Automated Thermochemistry for Heterogeneous Computational Chemistry Data. *F1000Res* **2020**, *9*, 291, doi:10.12688/f1000research.22758.1.

22. Ditchfield, R. Self-Consistent Perturbation Theory of Diamagnetism. *Mol Phys* **1974**, *27*, 789–807, doi:10.1080/00268977400100711.

23. Tantillo DJ Chemical Shift Repository. Http://Cheshirenmr. Info/Instr Uctions.Htm/.

24. Kamel, M.; Mohammadi, M.; Mohammadifard, K.; Mahmood, E.A.; Poor Heravi, M.R.; Heshmati J.M., A.; Hossaini, Z. Comprehensive Theoretical Prediction of the Stability and Electronic Properties of Hydroxyurea and Carmustine Drugs on Pristine and Chitosan-Functionalized Graphitic Carbon Nitride in Vacuum and Aqueous Environment. *Vacuum* **2023**, *207*, 111565, doi:10.1016/j.vacuum.2022.111565.
